# C6 Hydroxymethyl-Substituted Carbapenem MA-1-206 Inhibits the Major *Acinetobacter baumannii* Carbapenemase OXA-23 by Impeding Deacylation

**DOI:** 10.1128/mbio.00367-22

**Published:** 2022-04-14

**Authors:** Nichole K. Stewart, Marta Toth, Maha A. Alqurafi, Weirui Chai, Thu Q. Nguyen, Pojun Quan, Mijoon Lee, John D. Buynak, Clyde A. Smith, Sergei B. Vakulenko

**Affiliations:** a Department of Chemistry and Biochemistry, University of Notre Dame, Notre Dame, Indiana, USA; b Department of Chemistry, Southern Methodist University, Dallas, Texas, USA; c Mass Spectrometry and Proteomics Facility, University of Notre Dame, Notre Dame, Indiana, USA; d Stanford Synchrotron Radiation Lightsource, Stanford University, Menlo Park, California, USA; e Department of Chemistry, Stanford University, Stanford, California, USA; Louis Stokes Veterans Affairs Medical Center

**Keywords:** β-lactamase, OXA-23, *Acinetobacter*, inhibitor, carbapenem, crystal structure, catalytic mechanism, antibiotic resistance

## Abstract

Acinetobacter baumannii has become a major nosocomial pathogen, as it is often multidrug-resistant, which results in infections characterized by high mortality rates. The bacterium achieves high levels of resistance to β-lactam antibiotics by producing β-lactamases, enzymes which destroy these valuable agents. Historically, the carbapenem family of β-lactam antibiotics have been the drugs of choice for treating A. baumannii infections. However, their effectiveness has been significantly diminished due to the pathogen’s production of carbapenem-hydrolyzing class D β-lactamases (CHDLs); thus, new antibiotics and inhibitors of these enzymes are urgently needed. Here, we describe a new carbapenem antibiotic, MA-1-206, in which the canonical C6 hydroxyethyl group has been replaced with hydroxymethyl. The antimicrobial susceptibility studies presented here demonstrated that this compound is more potent than meropenem and imipenem against A. baumannii producing OXA-23, the most prevalent CHDL of this pathogen, and also against strains producing the CHDL OXA-24/40 and the class B metallo-β-lactamase VIM-2. Our kinetic and mass spectrometry studies revealed that this drug is a reversible inhibitor of OXA-23, where inhibition takes place through a branched pathway. X-ray crystallographic studies, molecular docking, and molecular dynamics simulations of the OXA-23-MA-1-206 complex show that the C6 hydroxymethyl group forms a hydrogen bond with the carboxylated catalytic lysine of OXA-23, effectively preventing deacylation. These results provide a promising strategy for designing a new generation of CHDL-resistant carbapenems to restore their efficacy against deadly A. baumannii infections.

## INTRODUCTION

Acinetobacter baumannii is a rapidly emerging Gram-negative ESKAPE pathogen (*Enterococcus faecium*, *Staphylococcus aureus*, *Klebsiella pneumoniae*, *Acinetobacter baumannii*, *Pseudomonas aeruginosa*, and *Enterobacter* species) which the CDC has deemed an urgent antibiotic resistance threat (1). It is typically responsible for ventilator-associated pneumonia, but may also cause bacteremia, endocarditis, meningitis, and wound, burn, and urinary tract infections (2). Until recently, carbapenems have been successfully used as last-resort antibiotics for treating such infections. However, over time, A. baumannii has developed exceptionally high levels of antibiotic resistance to these drugs, which, combined with its ability to persist for long periods on surfaces, have rendered this organism a major cause of hospital-acquired infections worldwide (3–7). Such multidrug-resistant A. baumannii (MDR*Ab*) and extensively resistant A. baumannii (XDR*Ab*) are extremely difficult to treat, resulting in high mortality rates. Currently, colistin or tigecycline is often used as a last-line therapy. However, colistin is nephrotoxic, and increased numbers of colistin- and tigecycline-resistant A. baumannii isolates (known as pandrug-resistant A. baumannii, PDR*Ab*) are being reported worldwide (8–11).

A. baumannii achieves resistance to β-lactam antibiotics through the production of β-lactamases, which can be exacerbated by reduced penetration due to porin modifications, upregulated efflux, and the ability to form biofilms (12–14). Although selected strains of A. baumannii produce β-lactamases of all four Ambler classes, this pathogen is most recognized for its ability to produce carbapenem-hydrolyzing class D β-lactamases (CHDLs), which render the pathogen resistant to the last-line carbapenem antibiotics. Among them, the OXA-23-like carbapenemases are most widespread and have been detected in up to 83% of A. baumannii clinical isolates (14). Alarmingly, coinfection with carbapenem-resistant A. baumannii strains (CR*Ab*) has now been identified in increasingly large numbers of patients hospitalized with SARS-CoV-2 (15).

Widespread carbapenem resistance in A. baumannii and other clinically important bacterial pathogens highlights the urgency of developing new carbapenem antibiotics resistant to inactivation by CHDLs. The carbapenem scaffold was discovered almost 50 years ago, in 1976, and its antibacterial activity was optimized to preserve breadth of both spectrum and stability in the class A and C β-lactamases of the 20th century (16). This has led to the current commercial carbapenems (Fig. S1 in the supplemental material), which vary structurally at the C2 position. In addition, a C1β-methyl group was introduced, which improves stability against human renal dehydropeptidase (DHP-1) compared to the first clinical carbapenem, imipenem (17). The scaffold has not been significantly altered in the past 2 decades, apart from modifications at the C2 position. However, a recent report has demonstrated that modification of a non-C2-position on the carbapenem scaffold (an α-face alkyl group at C5) can produce carbapenem antibiotics with significantly improved *in vitro* activity against resistant mycobacterial pathogens (18).

10.1128/mbio.00367-22.6 FIG S1 Current commercial carbapenem antibiotics. Download FIG S1, TIF file, 0.9 MB. Copyright © 2022 Stewart et al. 2022 Stewart et al. https://creativecommons.org/licenses/by/4.0/ This content is distributed under the terms of the Creative Commons Attribution 4.0 International license.

The serine β-lactamases (Ambler classes A, C, and D) all hydrolyze β-lactam compounds via a two-step mechanism comprising an acylation step (whereby a covalent bond is formed between the catalytic serine side chain and substrate) followed by deacylation to release an inactive product. The carbapenem C6 hydroxyethyl substituent has played a major role in stabilizing the antibiotic against deacylation in the class A and C β-lactamases through different mechanisms (19). These include the steric hindrance provided by the group, which forces the β-lactam C7 carbonyl oxygen to flip out of the oxyanion hole, and the formation of a hydrogen bond between the hydroxyethyl group and deacylating water, which decreases the water molecule’s nucleophilicity, thus stabilizing the acyl enzyme (20–22). In the class D enzymes, the acylation step requires activation of the catalytic serine by an adjacent lysine residue post-translationally modified by carboxylation; this same carboxylated lysine is also involved in activating a water molecule, which attacks the acyl bond during the deacylation step (23, 24). The C6 hydroxyethyl group of carbapenems is directed toward the carboxylated lysine pocket and, as a result, blocks access of water, thus preventing deacylation (22). Recent structural analyses have demonstrated that OXA carbapenemases have evolved the ability to hydrolyze carbapenem antibiotics by allowing ingress of the deacylating water to the active site. They accomplish this by opening a transient channel formed by the movement of one of two conserved hydrophobic surface residues (most commonly Leu and Val), which form a hydrophobic cap separating the active site from an internal pocket containing the carboxylated lysine residue (22, 25–27). Conversely, a recent report indicates this same C6-side chain hydroxyl can function as an intramolecular nucleophile, attacking the acyl-enzyme carbonyl carbon of carbapenem antibiotics, leading to formation of a β-lactone, thereby accelerating β-lactamase-mediated degradation (28, 29). This knowledge of the molecular mechanisms for the hydrolysis of carbapenems by class D carbapenemases allows us to envision new strategies to develop novel CHDL-resistant carbapenems. Such strategies include the following: (i) modification of the canonical C6 substituent, which would prevent opening of the channel for access by the deacylating water, (ii) displacement of the carboxylated lysine, and (iii) the creation of a strong hydrogen-bonding interaction with the carboxylated lysine to prevent activation of the deacylating water. To combat the growing problem of CHDL-mediated carbapenem resistance in A. baumannii and restore the utility of this valuable class of antibiotics, we evaluated the interaction of the C6-substituted hydroxymethyl carbapenem antibiotic MA-1-206 with OXA-23 by performing microbiological, kinetic, mass spectrophotometric, and structural studies.

## RESULTS AND DISCUSSION

### Design of MA-1-206.

**FIG 1:**
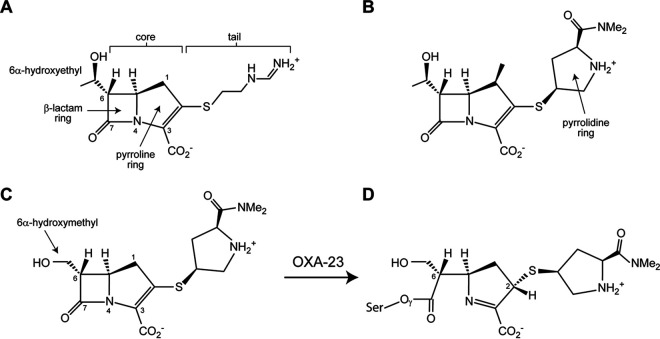
Structures of carbapenems. (A) Imipenem, with β-lactam ring, pyrroline ring, 6α-hydroxyethyl group, and core and tail regions indicated. Atom numbering is shown. (B) Meropenem, with pyrrolidine ring of the tail indicated. (C) MA-1-206. The tail group is the same as meropenem, but the core resembles imipenem (with no methyl group at C1), although it has the smaller α-hydroxymethyl group at C6. (D) Hydrolysis of MA-1-206 by the class D β-lactamase OXA-23 opens the β-lactam ring between N4 and C7 and results in a covalent acyl-enzyme intermediate as the Δ^1^ tautomer in an *S* configuration at C2.

The detailed protocol for MA-1-206 synthesis is described in the supplementary information (Text S1). Structurally, this compound is similar to both meropenem and imipenem (Fig. 1); its core structure is identical to that of imipenem, while the tail group at the C2 position is identical to that of meropenem. However, unlike any of the currently used carbapenems, MA-1-206 has a hydroxymethyl group at the C6 position instead of the hydroxyethyl group of classical carbapenems. Early studies of analogous desmethyl carbapenems have indicated broad-spectrum activity approximately equivalent to that of carbapenems possessing the usual carbapenem C6 hydroxyethyl substituent, but this modification has not been previously evaluated against strains of Acinetobacter or other organisms which produce CHDLs (30, 31).

10.1128/mbio.00367-22.1 TEXT S1 Procedures for synthesis of MA-1-206. Download Text S1, DOCX file, 0.4 MB. Copyright © 2022 Stewart et al. 2022 Stewart et al. https://creativecommons.org/licenses/by/4.0/ This content is distributed under the terms of the Creative Commons Attribution 4.0 International license.

### Antibiotic susceptibility testing.

To evaluate the microbiological activity of MA-1-206, we measured its minimum inhibitory concentrations (MICs) against our collection of A. baumannii CIP 70.10 strains producing various β-lactamases from a shuttle vector (22). These include clinically important carbapenemases of various classes that have been identified in A. baumannii with variable frequencies (CHDLs OXA-23, OXA-24/40, OXA-48, and OXA-58; class A enzymes KPC-6 and GES-5; metallo-β-lactamases [MBLs] NDM-1 and VIM-2). The collection also includes strains producing the intrinsic class C β-lactamase of A. baumannii, ADC-1, and the most common narrow-spectrum class A enzyme, TEM-1. We first evaluated the activity of MA-1-206 against A. baumannii CIP 70.10 expressing the most clinically widespread and important CHDL, OXA-23. We found that the compound was 8- and 4-fold more active than meropenem and imipenem, respectively (Table 1). Our results demonstrated that MA-1-206 was also 8- and 4-fold more active than the other two carbapenems against A. baumannii producing the MBL VIM-2. In addition, we observed that MA-1-206 had a 2- to 4-fold lower MIC against the strain producing OXA-24/40. For the A. baumannii strains expressing the remaining carbapenemases (except GES-5, where the MIC of the compound was 2- to 4-fold higher), the MICs of MA-1-206 were the same as those of meropenem. The compound also had potent activity against the class C ADC-1-producing strain; however, its MIC against the strain expressing TEM-1 was 16- and 32-fold higher than those of meropenem and imipenem, respectively, indicating that structural differences with the two other carbapenems rendered the compound more susceptible to hydrolysis by this enzyme. Finally, the MIC of MA-1-206 against the parental strain, which does not produce any β-lactamases from the shuttle vector, was the same as that of imipenem and 2-fold lower than that of meropenem, suggesting that the modification at the C6 position did not diminish the compound’s ability to penetrate A. baumannii cells and/or inhibit their penicillin-binding proteins.

**TABLE 1:** MICs (μg/mL) of β-lactams against A. baumannii CIP 70.10 expressing various β-lactamases

Enzyme	Antibiotic MICs (μg/mL)		
	Imipenem	Meropenem	MA-1-206
None^*a*^	0.25	0.5	0.25
OXA-23	32	64	8
OXA-24/40	64	128	32
OXA-48	16	16	16
OXA-58	8	4	4
KPC-6	32	256	256
GES-5	16	32	64
NDM-1	32	128	128
VIM-2	32	64	8
ADC-1	0.25	0.5	0.5
TEM-1	0.25	0.5	8

a Parental A. baumannii CIP 70.10 strain that does not produce any β-lactamase from the shuttle vector.

We next evaluated the MICs of MA-1-206 against 45 genotypically characterized multidrug-resistant A. baumannii isolates from the CDC & FDA Antibiotic Resistance (AR) Isolate Bank. The observed MIC values for meropenem, imipenem, and MA-1-206 against these clinical isolates closely mimicked those observed with our collection of strains, where β-lactamases were expressed from the shuttle vector. We observed the largest reduction in MICs with the isolates (*n* = 31) harboring the genes for the OXA-23 and/or OXA-24/40 carbapenemases, where the MICs of MA-1-206 were up to 16- and 8-fold lower than those of meropenem and imipenem, respectively (Table 2). For all other isolates, the MICs of the compound were similar to those of meropenem. Collectively, our MIC results demonstrated that the structural changes of MA-1-206 versus meropenem and imipenem significantly improved the compound’s ability to evade inactivation by the most clinically important A. baumannii CHDL, OXA-23. In addition, the compound is also more active against the CHDL OXA-24/40 and the MBL VIM-2.

**TABLE 2:** MICs (μg/mL) of β-lactams against A. baumannii clinical isolates

Enzyme^*a*^	Antibiotic MICs (μg/mL)			No. of strains
	Imipenem	Meropenem	MA-1-206	
OXA-23	16–64	32–128	8–16	22
OXA-23, OXA-24/40	64	128	16	1
OXA-24/40	64–128	128–256	32–64	8
OXA-58	8	8	8	2
OXA-72	64–128	256–512	≥128	8
NDM-1	64–256	128–512	≥128	3
NDM-1, OXA-23	128	256	≥128	1

a All strains encode OXA-51 (such as OXA-65, -66, -69, -82, -94, -100, -203, and -223) and ADC derivatives. Twenty strains encode TEM-type and two encode PER-7 β-lactamases.

### Evaluation of kinetics.

**FIG 2:**
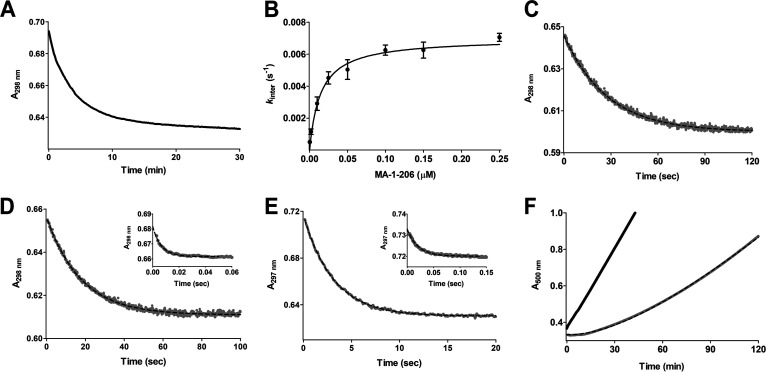
Kinetics of the interaction between OXA-23 and carbapenems. (A) Representative progress curve showing inhibition of 1 μM OXA-23 by MA-1-206. (B) Plot of *k*_inter_ as a function of MA-1-206 concentration. (C) Time course for acylation of OXA-23 by MA-1-206. Black dashed line represents the best fit of the data to Equation 5. (D) Time course for acylation of OXA-23 by meropenem. The faster phase of acylation with a 5-fold excess of enzyme is shown as an inset. Black dashed lines represent the best fit of the data to Equation 6. (E) Time course for acylation of OXA-23 by imipenem. The faster phase of acylation with a 5-fold excess of enzyme is shown as an inset. Black dashed lines represent the best fit of the data to Equation 6. (F) Progress curves for reactivation of OXA-23 following incubation in the presence (gray line) and absence (black line) of MA-1-206. Black dashed line represents the best fit of the data to Equation 7.

**FIG 3:**
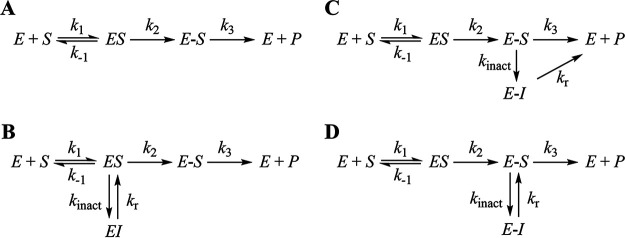
Potential minimal enzymatic reaction pathways for turnover of meropenem and imipenem by OXA-23 (A) or inhibition of the enzyme by MA-1-206 (B to D). *E* is the enzyme, *S* is the carbapenem, *ES* is the noncovalent Michaelis enzyme-substrate complex, *EI* is the noncovalent Michaelis enzyme-inhibitor complex formed after the initial 11 turnovers, *E-S* is the covalent enzyme-substrate complex, *E-I* is the covalent enzyme-inhibitor complex formed after the initial 11 turnovers, and *P* is the hydrolyzed carbapenem. In panel B, the inhibited complex is formed from the *ES* complex, while in panels C and D it is formed from the *E-S* complex. In panel C, the *E-I* complex is capable of deacylation, while in panel D it must revert back to the active *E-S* species prior to deacylation.

To evaluate the mechanism of interaction between MA-1-206 and OXA-23, the most predominant A. baumannii CHDL, we performed detailed kinetics studies. Because such experiments require knowledge of the molar extinction coefficient of the compound, we measured this parameter by monitoring the decrease in absorbance upon hydrolysis of the β-lactam ring at 298 nm, where the maximum change was observed. Next, we monitored whether there was an observable reaction between MA-1-206 and OXA-23 by using a continuous absorbance assay (with two different enzyme concentrations, 0.5 and 1 μM) under steady-state conditions and found that in the presence of OXA-23, progress curves showed that there was a decrease in absorbance, indicating that OXA-23 is able to hydrolyze the compound (representative curve for 1 μM is shown in Fig. 2A). The progress curves were biphasic, with a gradual decrease in the initial velocity over time. From these curves, we calculated the amount of compound hydrolyzed during the faster phase of the reaction by using the molar extinction coefficient and the change in absorbance, and found that 10.6 molecules of MA-1-206 per each molecule of OXA-23 were hydrolyzed before the rate of the reaction dramatically decreased. This constitutes only 5.3% or 10.6% of the total 100 μM of the compound present in the reaction mixture, when 0.5 or 1 μM enzyme was used, respectively. From this point on, we observed that MA-1-206 was still being hydrolyzed, albeit at a very slow constant rate, while the vast majority (∼90% to 95%) of the compound was still intact, indicating that the compound had transitioned to a very poor substrate, which could also be characterized as a reversible inhibitor (32). This is in stark contrast to the kinetics reported for both meropenem and imipenem; they are both moderately good substrates of OXA-23 and are fully hydrolyzed by the enzyme (Fig. 3A) (22, 23). We also evaluated the partition ratio (*r* = *k*_cat_/*k*_inact_) for OXA-23 and MA-1-206, which describes the number of inhibitor molecules that are turned over before an enzyme is inactivated and provides a measure of the inactivation efficiency of a compound. The lower the partition ratio, the better the inhibition potency of a compound. Our results yielded a partition ratio of 12 ± 1 (Table 3), which indicates that, on average, 11 molecules of MA-1-206 were hydrolyzed prior to inhibition of OXA-23 by the 12th molecule. This is in close agreement with our initial calculation based on reaction progress curves, which showed that 10.6 molecules had been hydrolyzed once the enzyme was inhibited.

**TABLE 3:** Kinetic parameters for the interaction between OXA-23 and carbapenems

Parameter	Antibiotic		
	Imipenem	Meropenem	MA-1-206
*k*_cat_ (s^−1^)	0.35 ± 0.01	0.068 ± 0.001	0.046 ± 0.001
*K*_s_ or *K*_i_ (nM)	190 ± 20	60 ± 10	26 ± 2
*k*_cat_/*K*_s_ or *k*_cat_/*K*_i_ (M^−1^s^−1^)	(1.8 ± 0.2) × 10^6^	(1.1 ± 0.2) × 10^6^	(1.8 ± 0.1) × 10^6^
*k*_2 fast_ (s^−1^)	≥330	≥330	
*k*_2 slow_ (s^−1^)	0.37 ± 0.01	0.056 ± 0.001	0.048 ± 0.001^*a*^
*k*_inact_ (s^−1^)	NA^*b*^	NA	0.0070 ± 0.0002
inactivation half-life, *t*_1/2_ (min)	NA	NA	1.7 ± 0.1
*K*_I_ (nM)	NA	NA	8.5 ± 1.1
*k*_inact_/*K*_I_ (M^−1^s^−1^)	NA	NA	(4.8 ± 0.7) × 10^5^
*k*_3_ (s^−1^)	0.35^*c*^^,^^*d*^, 6.5 ± 0.3^*c*^	0.12 ± 0.01	1.1 ± 0.1^*c*^
*k*_r_ (s^−1^)	NA	NA	0.00043 ± 0.00001
residence time (min)	0.048^*e*^, 0.0026 ± 0.0001	0.14 ± 0.01	0.015 ± 0.002^*f*^, 39 ± 1
partition ratio	NA	NA	12 ± 1

a For MA-1-206, the reaction was monophasic, characterized by only one *k*_2_ value.

b NA, not applicable.

c Calculated using Equation 8.

d The first value was calculated using *k*_2 fast_, while the second was calculated using *k*_2 slow_.

e The first value was calculated using *k*_3_ resulting from *k*_2 fast_, while the second was calculated using *k*_3_ resulting from *k*_2 slow_.

f The first value was calculated using *k*_3_, while the second was calculated using *k*_r_.

We next measured the effectiveness of inactivation of OXA-23 by MA-1-206 during the slow phase of the reaction by measuring the second-order rate constant, *k*_inact_/*K*_I_, which is often used to describe the inhibitory potency of a compound. A plot of *k*_inter_ values versus MA-1-206 concentration gave a hyperbolic curve (Fig. 2B), indicating that inactivation takes place after the formation of the noncovalent Michaelis complex (33) (as shown in Fig. 3B–D). The *k*_inact_/*K*_I_ value was found to be (4.8 ± 0.7) × 10^5^ M^−1^s^−1^ (Table 3), showing that OXA-23 is inactivated by MA-1-206 with relatively high efficiency. From this plot, we were also able to evaluate the individual parameters *k*_inact_ and *K*_I_, which are the first-order rate constant for inactivation and the apparent dissociation constant (analogous to *K*_m_ for enzyme substrates) that describes the concentration of inhibitor required to give one-half the rate of *k*_inact_, respectively. The *k*_inact_ value was found to be only 0.0070 ± 0.0002 s^−1^, which would result in an inactivation half-life of 1.7 ± 0.1 min or one inactivation event per 2.4 ± 0.2 min. The measured *K*_I_ value was also very low (8.5 ± 1.1 nM), revealing that the very high apparent affinity of OXA-23 for MA-1-206 is mostly responsible for the high inactivation efficiency. We further characterized the strength of the interaction between OXA-23 and MA-1-206 by measuring the dissociation constant, *K_i_*, which describes the true affinity between the enzyme and inhibitor in the noncovalent Michaelis complex. The *K_i_* value was measured to be 26 ± 2 nM (Table 3). This affinity is 7- and 2-fold higher than those we measured for imipenem and meropenem, respectively (23), demonstrating that the structural changes in MA-1-206 improved its affinity for OXA-23. Both the *K_i_* and *K*_I_ values are well below the MICs of MA-1-206 against the A. baumannii strains producing OXA-23 (8 to 16 μg/mL, or 20 to 40 μM) (Tables 1 and 2), indicating that at these concentrations, the compound would completely saturate OXA-23.

To gain deeper insights into the turnover kinetics during the fast phase of the reaction, when MA-1-206 acts as a substrate prior to its inhibition of OXA-23, we performed pre-steady-state kinetic experiments. We first conducted single-turnover experiments with a 5-fold excess of OXA-23 over MA-1-206, meropenem, or imipenem to measure the acylation rate constant, *k*_2_, which describes the maximum rate of acylation as shown in Fig. 3 (for MA-1-206, this is the first 11 turnovers during the fast phase of the reaction). For acylation of OXA-23 by MA-1-206, as expected, we observed an exponential decay in the absorbance signal (Fig. 2C) which, when fit to Equation 5, yielded a *k*_2_ value of 0.048 ± 0.001 s^−1^ (Table 3). This rate did not increase upon using a higher enzyme concentration with a 20-fold molar excess over the compound, indicating that saturation had been reached. In contrast to the monophasic acylation reaction observed for MA-1-206, for the other two carbapenems, this reaction was biphasic, with an initial faster phase (*k*_2 fast_) comprising 10% or 30% of the total reaction for imipenem and meropenem, respectively, which was followed by a much slower phase (*k*_2 slow_) (Fig. 2D and E). However, the faster phase for imipenem and meropenem could no longer be observed when the concentration of OXA-23 was increased to 20-fold over that of these substrates, indicating that *k*_2 fast_ must be ≥330 s^−1^, which exceeded the detection limit of the instrument. The *k*_2 slow_ values for meropenem and imipenem were measured to be 0.056 ± 0.001 s^−1^ and 0.37 ± 0.01 s^−1^, respectively, (Table 3), and did not increase with a 20-fold molar excess of enzyme. These results showed that the acylation rate *k*_2_ of MA-1-206 for OXA-23 is very similar to *k*_2 slow_ of meropenem and 8-fold slower than that of imipenem. However, compared to the *k*_2 fast_ value for meropenem and imipenem, acylation of the compound is much slower, by at least 6,900-fold. The biphasic acylation kinetics we observed with meropenem and imipenem have previously been reported for the class A enzymes GES-1, GES-2, GES-5, and KPC-2 with various carbapenems, and were postulated to be a result of different conformers of carbapenems and/or different enzyme conformations (34–36). In support of the latter, recent molecular dynamics simulations with KPC-2 have suggested the existence of different enzyme conformations with differing catalytic competencies (37). For CHDLs, pre-steady-state kinetics have been reported for OXA-48 and the OXA-48-like enzyme OXA-163 (38). In this study, the authors did not observe biphasic kinetics for acylation of the enzyme with meropenem and imipenem and found that the *k*_2_ values ranged from 95 to 300 s^−1^, similar to the *k*_2 fast_ values we measured for these carbapenems for the faster phase of acylation of OXA-23.

To further evaluate the kinetics during the fast phase of the reaction prior to inhibition of OXA-23 by MA-1-206, we calculated the deacylation rate constant, *k*_3_. This value can be calculated using Equation 8 when the *k*_2_ and *k*_cat_ values are known. As we had already measured the *k*_2_ value, we proceeded to determine *k*_cat_ by using the initial velocities observed in our progress curves (Fig. 2A) (39). In the initial phase of the reaction, almost all of the enzyme is active, and the amount of inactivated enzyme is negligible. Because the concentration of MA-1-206 in these reactions was more than 3,800-fold above the *K_i_* value, OXA-23 would be saturated under these conditions, and the observed rate (0.046 ± 0.001 s^−1^) would be equivalent to *k*_cat_. This value is very similar to the *k*_cat_ value we previously measured for meropenem and is 8-fold below that of imipenem (Table 3). Of note, the catalytic efficiency (*k*_cat_/*K_i_*) of OXA-23 for MA-1-206 during the first 11 turnovers was identical to the *k*_cat_/*K_s_* value for imipenem and almost 2-fold higher than that of meropenem. These data show that prior to inhibition, the compound is turned over with relatively high efficiency, similar to that of the substrates meropenem and imipenem. Using the *k*_cat_ and *k*_2_ values for MA-1-206, we calculated the deacylation rate constant *k*_3_ for the fast phase of the reaction during the first 11 turnovers and found it to be 1.1 ± 0.1 s^−1^. We also determined *k*_3_ values for imipenem and meropenem. Because the deacylation rate for imipenem was too fast to be detected, it was also calculated using Equation 8 with our measured *k*_2_ and *k*_cat_ values. Since we measured *k*_2_ rate constants for both the fast and slow phases of acylation for imipenem, *k*_3_ was calculated to be either 0.35 s^−1^ (if using *k*_2 fast_) or 6.5 ± 0.3 s^−1^ (if using *k*_2 slow_). For meropenem, the deacylation reaction was slow enough that it could be measured using the jump dilution method, and *k*_3_ was found to be 0.12 ± 0.01 s^−1^ (Table 3). Comparison of the deacylation rate of MA-1-206 for the first 11 turnovers with those of imipenem and meropenem showed that the compound deacylates from OXA-23 9-fold faster than meropenem, but either 3-fold faster or 6-fold slower than imipenem (compared to the *k*_3_ values of imipenem calculated using *k*_2 fast_ or *k*_2 slow_, respectively). In the study of OXA-48 and OXA-163, the authors found that the *k*_3_ values for meropenem and imipenem ranged from 0.011 to 3.0 s^−1^ and determined that deacylation is the rate-limiting step for these two enzymes (38). For OXA-23, deacylation would also be rate-limiting, but only if the faster phase of acylation (dictated by *k*_2 fast_) for these two carbapenems was relevant for the steady state. Unfortunately, we could not definitively determine which phase of acylation for meropenem and imipenem is relevant for the steady state; for meropenem, using either *k*_2 fast_ or *k*_2 slow_ and *k*_3_ with Equation 8 gave calculated *k*_cat_ values that were similar to the measured value; for imipenem, *k*_3_ was too fast to be detected. For MA-1-206, we found that *k*_2_ was slower than *k*_3_, indicating that acylation is the rate-limiting step of the fast phase of the reaction (for the first 11 turnovers) prior to inhibition of OXA-23.

As we observed that the reaction between OXA-23 and MA-1-206 significantly slowed after the initial faster phase of hydrolysis, indicating that the enzyme was being inhibited, we evaluated whether its activity could be restored in the absence of the compound. To accomplish this, we measured the enzyme’s activity (defined as *k*_r_, Fig. 3B–D) by performing jump dilution experiments. These experiments showed that reactivation occurred at a slow rate (*k*_r_ = 0.00043 ± 0.00001 s^−1^) (Fig. 2F and Table 3), confirming that after inhibition, the interaction between OXA-23 and MA-1-206 is reversible. This *k*_r_ value is 2,600-fold lower than the *k*_3_ value of MA-1-206 prior to inhibition, which indicates that deacylation or reactivation of the inhibited complex occurs much more slowly than deacylation in the first 11 turnovers before inhibition. This rate constant is also 16-fold lower than the measured *k*_inact_ value (Table 3), revealing that *k*_r_ is the rate-limiting step of the slow phase of the reaction (Fig. 3B–D). This slow reactivation rate of the inhibited complex would result in a residence time of MA-1-206 of 39 ± 1 min, which is almost 2-fold longer than the doubling time for the A. baumannii CIP 70.10 strain (22 ± 0.4 min) (40). Therefore, following inhibition, OXA-23 would likely not regain activity for the entire growth cycle of the bacterium. The residence time of MA-1-206 during the slow phase of the reaction is 280- to 15,000-fold longer than those of meropenem and imipenem, demonstrating that substitution of the hydroxyethyl group with hydroxymethyl imparts stability to the compound against hydrolysis by OXA-23.

### Liquid chromatography/mass spectrometry analysis of products of enzymatic reaction.

Recent studies have shown that the carbapenem-class D enzyme acyl-enzyme complex can resolve not only through the classical hydrolytic mechanism, but also through the formation of a β-lactone, where the C6 hydroxyethyl group of the antibiotic attacks the ester carbonyl of the complex (28, 29). To determine whether any β-lactone is formed upon reaction of MA-1-206 with OXA-23, we analyzed the products from both the fast and slow phases of the reaction using liquid chromatography/mass spectrometry (LC/MS). For the fast phase, we set up a reaction between the compound and enzyme such that only four turnovers would take place to ensure that the majority of products would be formed prior to inhibition of the enzyme, which occurs on average after 11 turnovers. After incubation for 5 and 10 min, LC/MS analysis revealed that MA-1-206 had been entirely converted to the hydrolyzed product, and no β-lactone was detected (Fig. S2, control is shown in panel A, while the 10-min reaction is shown in panel B). To assess the slow phase of the reaction, we set up a reaction with a 15-fold molar excess of MA-1-206 over OXA-23, which was incubated for 1 h to ensure full inhibition of the enzyme. Next, excess compound was removed chromatographically (see Materials and Methods), and the acyl-enzyme complex was incubated for an additional 3 h to allow for product formation. Subsequent LC/MS analysis showed that, similar to what we observed during the fast phase of the reaction, only hydrolyzed MA-1-206 was present, and we did not detect any β-lactone (Fig. S2C). These data are in agreement with those of previous studies, which demonstrated that β-lactone is not formed upon reaction of OXA-23 with other carbapenems (imipenem and panipenem) that are also C1-unsubstituted (29).

10.1128/mbio.00367-22.7 FIG S2 Mass spectra of MA-1-206 and products from the enzymatic reaction with OXA-23. (A) MA-1-206 (theoretical *m/z* [M + H]^+^ of 356.1275) alone after passing through a molecular weight cut-off (MWCO) filter. (B) Products (theoretical *m/z* [M + H]^+^ for hydrolyzed MA-1-206 of 374.1380) of reaction of MA-1-206 with OXA-23 after 10 min of incubation. (C) Products of reaction of MA-1-206 with OXA-23 after inhibition of the enzyme, removal of excess compound, and 3 hrs of additional incubation. For experimental details, see Materials and Methods. Download FIG S2, TIF file, 2.8 MB. Copyright © 2022 Stewart et al. 2022 Stewart et al. https://creativecommons.org/licenses/by/4.0/ This content is distributed under the terms of the Creative Commons Attribution 4.0 International license.

Taken together, our kinetic and LC/MS experiments showed that the reaction between MA-1-206 and OXA-23 follows the hydrolytic mechanism and is characterized by biphasic progress curves, with a rapid exponential phase followed by a much slower linear phase (Fig. 2A). We observed that during the faster phase of the reaction, the concentration of product formed was greater than the concentration of OXA-23, indicating that initially, the compound acts as a substrate of OXA-23, and subsequently, after the initial 11 turnovers, the compound reversibly inhibits the enzyme. We also found that the *k*_inact_ value is less than the *k*_cat_ value. These findings all constitute evidence for a branched kinetic pathway, which has also been reported for various β-lactamases and the integral membrane protein BlaR1 with imipenem and other β-lactams (39, 41–44). Several different mechanisms can be envisioned to describe the reaction between MA-1-206 and OXA-23 (Fig. 3B–D). In all of these, the enzyme is progressively inhibited over the course of time corresponding on average to the first 11 rounds of turnover. The subsequent formation and resolution of the inhibited species can proceed via different pathways. First, inhibition can take place after formation of the noncovalent Michaelis complex (*ES*) to form a noncovalent inactivated complex (*EI*); subsequently, this complex slowly reverts back to reform the active *ES* complex, which can then proceed through acylation and deacylation (Fig. 3B). Second, inhibition can take place after formation of the acyl-enzyme species (*E-S*) to irreversibly form a covalent inactivated complex (*E-I*), which slowly deacylates to regenerate active enzyme (Fig. 3C). Lastly, inhibition can also take place after formation of the *E-S* species; however, in this case, the inactivated species can revert back to reform the active *E-S* complex, which subsequently deacylates (Fig. 3D). Finally, a more complex scenario can be envisioned, where branching occurs at more than one species. Of these scenarios, those shown in Fig. 3C and D have been most commonly reported (39, 42–44). However, it is difficult to distinguish between these pathways using kinetics alone. In all of these mechanisms, the branch could be a result of a chemical rearrangement of the noncovalent Michaelis complex or the covalent acyl-enzyme complex, a conformational change of the protein and/or substrate itself, or a combination of these events. From our kinetic experiments, we were able to rule out an alternative mechanism (not shown in Fig. 3) in which the free enzyme converts from an active to an inactive form, thereby inhibiting the reaction prior to binding of MA-1-206. If this were the case, we would have observed decreasing *k*_inter_ values in response to increasing MA-1-206 concentrations; however, our data showed increases in these values (Fig. 2B).

### Structural analysis of MA-1-206 binding.

**FIG 4:**
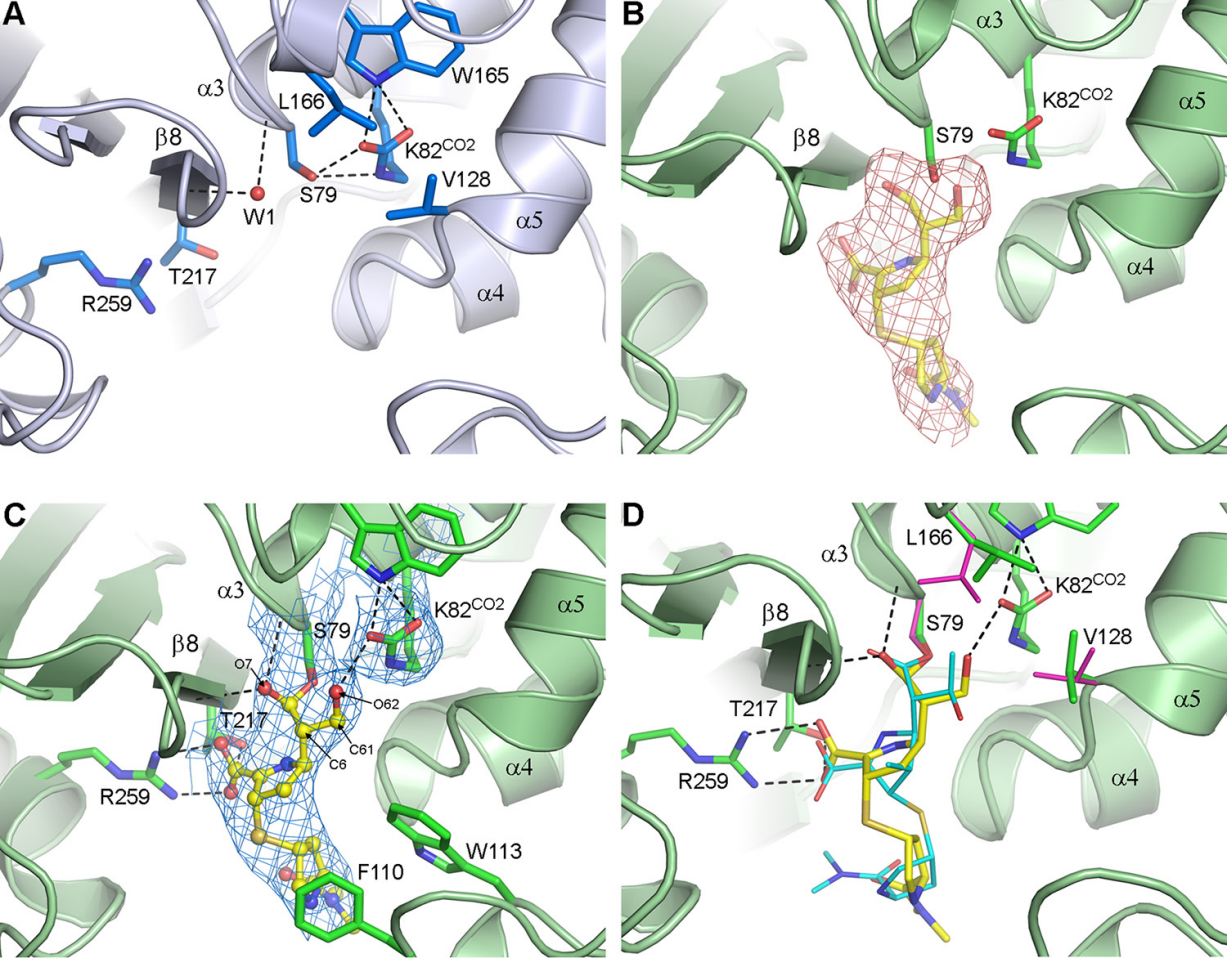
MA-1-206 binding to OXA-23. (A) Active site of apo-OXA-23 (PDB code 4JF6, light blue ribbons and sticks) showing the catalytic serine (Ser79) and the carboxylated lysine (Lys82^CO2^). Two juxtaposed aliphatic residues (Val128 and Leu166) form a hydrophobic cap which sequesters Lys82^CO2^ in an internal pocket. A water molecule (W1) is bound in the oxyanion hole at the N-terminus of helix α3. (B) Active site of OXA-23 (green ribbons and sticks) after 25 min incubation with the compound. Residual *F_o_-F_c_* density calculated after molecular replacement and prior to the addition of ligands to the model is shown (pink mesh, 3.0 σ). Location of the fully refined hydrolyzed MA-1-206 as the Δ^1^*S* tautomer at *t *=* *25 min is shown as semi-transparent yellow sticks. (C) Final 2*F_o_-F_c_* electron density (blue mesh, 1.0 σ) for the hydrolyzed compound at *t *=* *25 min. OXA-23 is shown as green ribbons and sticks, while MA-1-206 is shown as yellow balls and sticks. (D) Superposition of the OXA-23-meropenem complex (PDB code 4JF4; for clarity, only Val128 and Leu166 of OXA-23 are shown as magenta sticks, while meropenem is shown as thin cyan sticks) onto the *t *=* *25 min OXA-23-MA-1-206 complex (shown as yellow sticks and green ribbons and sticks, respectively). Hydrogen-bonding interactions are shown as black dashed lines.

To gain further insights into the interaction between OXA-23 and MA-1-206, we solved the X-ray crystal structure of their complex. The acylation of OXA-23 following addition of MA-1-206 was monitored at seven time points (Table S1 in the supplemental material). At all points, a single large piece of residual *F_o_-F_c_* electron density, calculated following molecular replacement, was observed in the enzyme active site, adjacent to the catalytic serine (Ser79). The apo-OXA-23 structure at pH = 7.0 (PDB code 4JF6) was used as the fully unacylated reference point, *t *=* *0 (Fig. 4A), and the longest soak time, *t *=* *25 min, was used as the fully acylated reference point (Fig. 4B). At this final time point, a complete MA-1-206 molecule could be readily built into the available density and refined using *phenix.refine* (Fig. 4C). The compound is covalently attached to the Ser79 side chain and anchored by six hydrogen bonds. The side chain of Ser79 rotates approximately 20° to form a covalent bond with MA-1-206, breaking the hydrogen-bonding interaction between the serine and the carboxylated lysine (Lys82^CO2^) initially present in apo-OXA-23 (Fig. 4A). The O7 carbonyl oxygen (formerly the β-lactam carbonyl of unhydrolyzed MA-1-206; Fig. 1C) is bound in the oxyanion hole, hydrogen bonded to the amide nitrogen atoms of Ser79 and Trp219 (Fig. 4C), where it displaces a water molecule bound at the same site in the apo-OXA-23 structure (W1, shown in Fig. 4A). The carboxylate moiety on the C3 atom of the pyrroline ring (Fig. 1C) makes three hydrogen-bonding interactions with two conserved residues, Arg259 and Thr217 (Fig. 4C). The arginine residue is conserved in the class D enzymes, and the threonine, on strand β8, is part of a universally conserved KTG serine β-lactamase fingerprint motif. On the opposite side of the pyrroline ring, the C6 hydroxymethyl group is directed into the carboxylated lysine pocket, where it makes a hydrogen-bonding interaction with the OQ2 atom of the fully carboxylated lysine (Fig. 4C and Table S1).

10.1128/mbio.00367-22.2 TABLE S1 Time points used for the acylation of OXA-23 by MA-1-206. Download Table S1, DOCX file, 0.01 MB. Copyright © 2022 Stewart et al. 2022 Stewart et al. https://creativecommons.org/licenses/by/4.0/ This content is distributed under the terms of the Creative Commons Attribution 4.0 International license.

Hydrolysis of the β-lactam ring of carbapenems leads to the formation of an intermediate in which the C2 atom of the pyrroline ring is initially *sp*^2^-hybridized. The sulfur atom is coplanar with the pyrroline ring, and this is known as the Δ^2^ tautomer. Subsequent isomerization of the double bond in the ring leads to a *sp*^3^-hybridized C2 carbon, giving the Δ^1^ tautomer in one of two enantiomeric forms, *S* and *R* (25). In the case of MA-1-206, the pyrroline ring of the acyl-enzyme intermediate observed at *t *=* *25 min is in the Δ^1^*S* tautomeric state (Fig. 1D). There is no evidence for the presence of either the Δ^2^ or the Δ^1^*R* tautomer, both of which have been observed in some structures of class D β-lactamase-carbapenem complexes (25, 45–49). The pyrrolidine tail projects away from the core of the molecule out of the active site in well-defined electron density (Fig. 4B), making hydrophobic interactions with the side chains of Phe110 and Trp113.

Superposition of the OXA-23-meropenem complex (PDB code 4JF4) onto the *t *=* *25 min complex shows that the pyrroline ring of MA-1-206 is tilted approximately 30° relative to the corresponding ring in meropenem (Fig. 4D). In the OXA-23-meropenem complex, the 6α-hydroxyethyl group is rotated such that the oxygen atom points away from the Ser79 and Lys82 side chains, and the terminal ethyl carbon pushes on the side chain of Leu166, one of the residues comprising the hydrophobic cap covering the carboxylated lysine in this enzyme. This results in a change to the leucine rotamer conformation relative to apo-OXA-23, which in earlier studies was deemed to be the “open” conformation (22). In the *t *=* *25 min MA-1-206 complex, this residue remains in the “closed” conformation, likely because there is no steric pressure on the leucine from the hydroxymethyl group. Instead, the Val128 side chain, the other residue which forms the hydrophobic cap, has rotated into a conformation (Fig. 4D) similar to the “open” conformation described for OXA-143 (26) and OXA-24/40 (25). This could occur spontaneously or in response to steric pressure from rotation of the hydroxyl group into the lysine pocket and the formation of the hydrogen-bonding interaction with the Lys82^CO2^.

Because MA-1-206 is a carbapenem derivative which resembles both meropenem and imipenem, in addition to comparing its complex with OXA-23 to that with meropenem (22), we also solved the structure of the OXA-23-imipenem complex for comparison. Imipenem was soaked for 2 min into pre-formed apo-OXA-23 crystals under the same conditions as MA-1-206. Residual 2*F_o_-F_c_* and *F_o_-F_c_* electron density was observed in the active site near Ser79 (Fig. S3A and B), indicative of full acylation, and the final 2*F_o_-F_c_* electron density for the imipenem is shown in Fig. S3C.

10.1128/mbio.00367-22.8 FIG S3 *In crystallo* soaking of imipenem into OXA-23. (A) Residual *F_o_-F_c_* electron density map (pink mesh, 2.5 σ) after soaking OXA-23 (light brown ribbons and orange sticks) crystals in an imipenem solution for 2 minutes. The unbiased map was calculated after molecular replacement and prior to the incorporation of the ligand into the model. (B) Residual 2*F_o_-F_c_* electron density map (blue mesh, 1.0 s) near the catalytic serine, Ser79, following imipenem soaking. Location of the refined imipenem from the final model is shown as gray semi-transparent sticks. (C) Ribbon representation of OXA-23 with bound imipenem. The final 2*F_o_-F_c_* electron density for the ligand and Lys82^CO2^ is shown as a blue mesh contoured at 1.0 σ. (D) Superposition of MA-1-206 (yellow sticks, at soak time *t *=* *25 min), imipenem (gray sticks), and meropenem (cyan sticks) bound to OXA-23. The 6α-hydroxyethyl groups of imipenem and meropenem are indicated, along with the 6α-hydroxymethyl group of MA-1-206. In panels A to C, hydrogen-bonding interactions are shown as black dashed lines. Download FIG S3, TIF file, 1.3 MB. Copyright © 2022 Stewart et al. 2022 Stewart et al. https://creativecommons.org/licenses/by/4.0/ This content is distributed under the terms of the Creative Commons Attribution 4.0 International license.

Similar to MA-1-206 and meropenem, imipenem binds such that the O7 carbonyl is anchored in the oxyanion hole by two hydrogen bonds, and the C3 carboxylate interacts with the side chains of Arg259 and Thr217. As was observed with meropenem and MA-1-206 in their acyl-enzyme complexes with OXA-23, the C2 carbon is *sp*^3^-hybridized, and the pyrroline ring adopts the Δ^1^*S* tautomer conformation. However, unlike with meropenem, the orientation of the ring is similar to that of MA-1-206, lacking the 30°-rotation observed in the OXA-23-meropenem complex (Fig. S3D). Subsequent modeling experiments with meropenem (data not shown) showed that its pyrroline ring cannot occupy the same orientation as observed in MA-1-206 and imipenem due to steric hinderance of its C1 methyl group with the carbonyl oxygen of Trp219. Thus, it is likely that the observed 30°-rotation of meropenem is caused by this steric constraint.

The pyrroline ring and the tail of imipenem are clearly defined; however, the 6α-hydroxyethyl group lacks strong density. Refinement places this group in an orientation which is slightly rotated relative to the orientation observed in the OXA-23-meropenem complex (Fig. S3D), and the difference in the pyrroline ring tilt of the two molecules results in the hydroxyl groups being in roughly the same position. Although the orientation of the imipenem 6α-hydroxyethyl group cannot be unequivocally defined given its weak density, this group would have some limited rotational degrees of freedom despite the close proximity of the Val128 and Leu166 side chains, which form the hydrophobic cap.

### *In crystallo* time-dependent binding of MA-1-206.

**FIG 5:**
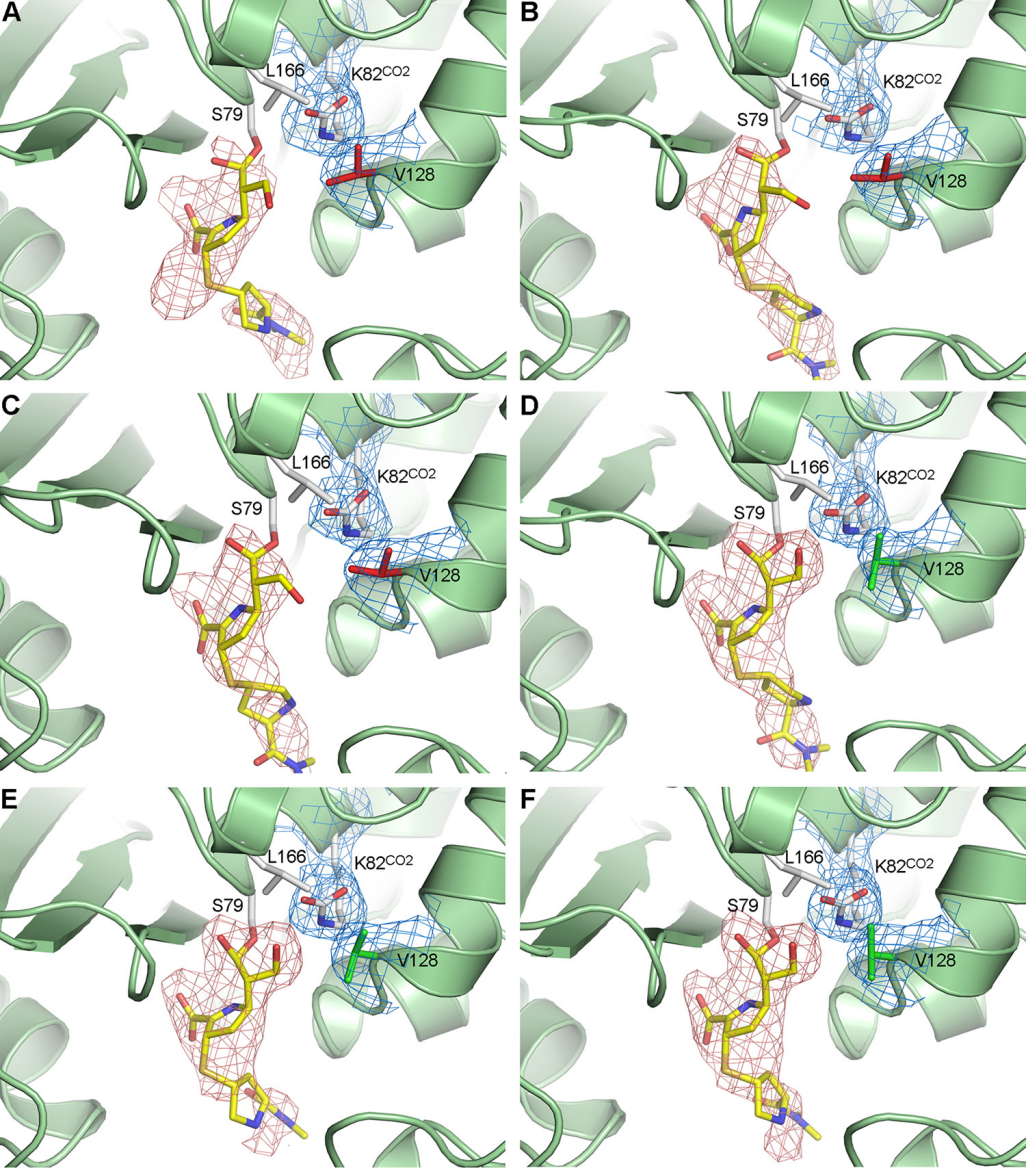
*In crystallo* snapshots of the OXA-23 active site upon MA-1-206 binding. (A) Time point *t *=* *30 s. (B) *t *=* *1 min. (C) *t *=* *2 min. (D) *t *=* *3 min. (E) *t *=* *5 min. (F) *t *=* *10 min. In all panels, residual *F_o_-F_c_* electron density calculated after molecular replacement and prior to the addition of ligands to the model is shown as a pink mesh contoured at 3.5 σ. Blue mesh represents the 2*F_o_*-*F_c_* electron density calculated after the first round of refinement in the presence of MA-1-206, and is shown for the Lys82^CO2^ and Val128 side chains. The carboxylate moiety on the lysine remains fully occupied throughout the course of the time-resolved experiment (Table S1). The Val128 side chain is colored red in panels A, B, and C, representing the “closed” side chain conformation, and is colored green in panels D, E, and F, representing the “open” conformation. The lack of density for the 6α-hydroxymethyl side group is evident in panels A, B, and C, whereas at time points *t *≥* *3 min, the density for this side group is clearly directed toward Lys82^CO2^.

Our time-dependent studies showed substantial density for the pyrroline ring, the sulfur atom, part of the pyrrolidine tail of MA-1-206, and the acyl bond with the catalytic serine (Ser79) from the earliest time point (*t *=* *30 s) onwards (Fig. 5 and Fig. S4). Much weaker density (only observable at low σ values) for the 6α-hydroxymethyl side group was seen for up to 2 min of observation, indicating that while a substantial amount of the acyl-enzyme OXA-23-MA-1-206 complex accumulates early in the reaction, the 6α-hydroxymethyl side group of MA-1-206 remains mobile for most molecules for at least the first 2 min. Refinement of the 30 s ≤ *t *≤* *2 min complexes shows that at these early time points, the hydroxyl group adopts an orientation pointing away from Ser79 and Lys82^CO2^ (Fig. 5A–C). In contrast, at time points 3 min ≤ *t *≤* *10 min, there is clear density for the hydroxymethyl group (Fig. 5D–F), and refinement of these three complexes invariably has the hydroxyl group within hydrogen-bonding distance of Lys82^CO2^. At the earliest time point, there is a small density lobe in the residual *F_o_-F_c_* map, suggesting that some MA-1-206 molecules may still be present as the Δ^2^ tautomer (Fig. 5A). However, at all later time points, conversion to the Δ^1^*S* tautomer is complete, and there is no evidence for the presence of the Δ^2^ tautomer (Fig. 5B–F).

10.1128/mbio.00367-22.9 FIG S4 *In crystallo* soaking of MA-1-206 into OXA-23 after 30 seconds. Stereoview of the OXA-23 active site (light green ribbons and green sticks) showing the 2*F_o_-F_c_* electron density (blue mesh contoured at 1 σ) for the catalytic serine (Ser79), the carboxylated lysine (K82^CO2^), and the bound MA-1-206 (yellow sticks). The unbiased map was calculated after molecular replacement and prior to the incorporation of the ligand into the model. Download FIG S4, TIF file, 2.5 MB. Copyright © 2022 Stewart et al. 2022 Stewart et al. https://creativecommons.org/licenses/by/4.0/ This content is distributed under the terms of the Creative Commons Attribution 4.0 International license.

Next, we calculated the occupancy of MA-1-206 and its hydroxymethyl group at all time points with *phenix.refine* using two methods: (i) applying and refining a single average group occupancy value to all MA-1-206 atoms, and (ii) fixing the occupancy of all atoms at 1.0 except the C61 and O62 atoms of the hydroxymethyl group (Table S1). At the three shortest time points (30 s ≤ *t *≤* *2 min), the average MA-1-206 occupancy was less than unity but was still well occupied (approximately 80%), as expected based upon the strength of the residual *F_o_-F_c_* density. This increased to nearly 100% for the time points greater than 3 min. In contrast, at the early time points (30 s ≤ *t *≤* *2 min), occupancy of the O62 hydroxyl atom was much lower (35 to 55%); however, it increased to 100% at the longer time points (Table S1). These data further confirm the increased mobility of the 6α-hydroxymethyl side group of MA-1-206 compared to the rest of the molecule at early time points of observation, and also confirm that the mobility of this group decreases once it becomes involved in a hydrogen-bonding interaction with the carboxylated lysine at *t *≥* *3 min. We also calculated the occupancy of the carboxylated lysine at all time points and found it to be fully occupied (Table S1).

**FIG 6:**
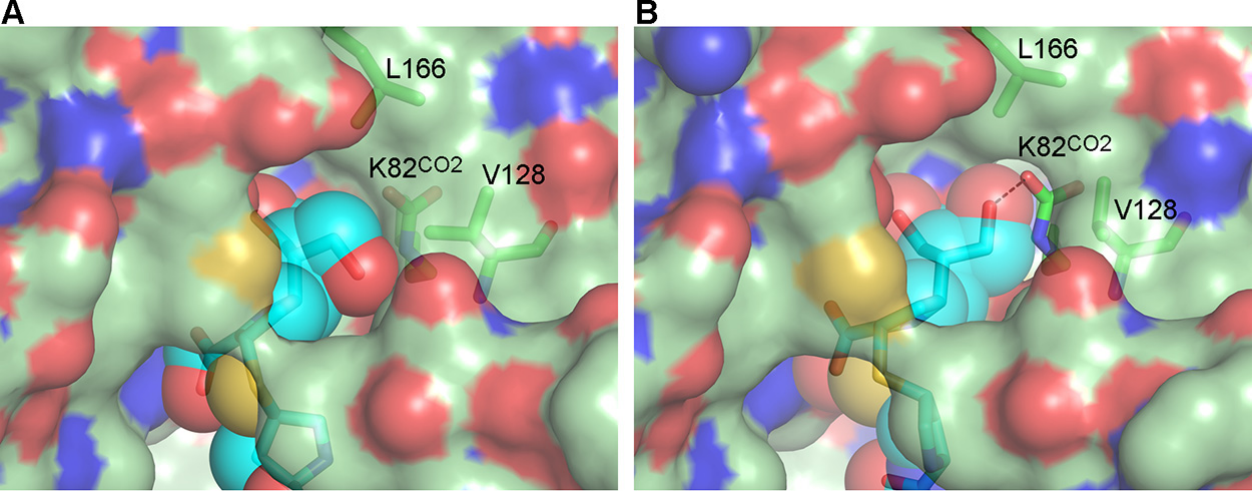
The hydrophobic cap in OXA-23. (A) Molecular surface in the active site of OXA-23 (light green semi-transparent surface and green sticks) after soaking for 30 s with MA-1-206 (cyan sticks and semi-transparent spheres). The side chains of Lys82^CO2^, Val128, and Leu166 are visible beneath the surface. (B) Molecular surface in the active site of OXA-23 (light green semi-transparent surface and green sticks) after soaking for 25 min with MA-1-206 (cyan sticks and semi-transparent spheres). MA-1-206 and the side chains of Lys82^CO2^, Val128, and Leu166 are visible beneath the surface. The hydrogen bond between the MA-1-206 6α-hydroxymethyl group and the Lys82^CO2^ is indicated by a dashed line.

At time points 30 s ≤ *t *≤* *2 min, both the Val128 and Leu166 side chains were in “closed” conformations, whereas at time points 3 min ≤ *t *≤* *25 min, the Val128 side chain transitioned to the “open” conformation during refinement. Calculation of the molecular surface inside the active site of the *t *=* *30 s complex shows that when these two residues are closed, there is a continuous surface over the carboxylated lysine (Fig. 6A). At the 3 min ≤ *t *≤* *25 min time points, the Val128 side chain was in the “open” rotamer configuration; however, the hydroxymethyl group of MA-1-206, which is hydrogen-bonded to the carboxylated lysine, seems to effectively plug this channel (Fig. 6B) and could prevent water from accessing the lysine pocket.

The time-dependent interaction of MA-1-206 with OXA-23 described above suggests that while acylation of Ser79 by the compound for the majority of enzyme molecules happens rapidly (≤30 s), formation of the hydrogen bond between the hydroxymethyl of the compound and the carboxylated lysine residue occurs more slowly, over the course of several minutes. During this time, the hydroxyl group of most acylated MA-1-206 molecules may be freely rotating, and the initial faster phase of hydrolysis is most likely occurring (Fig. 2A). This implies that a water molecule enters into the vicinity of the carboxylated lysine, where it becomes activated; this requires the channel to the external milieu to be opened to allow the ingress of water, since there is no other entrance into the carboxylated lysine pocket (22, 27). In order for this to happen, one or both of the residues comprising the hydrophobic cap (Val128 and Leu166) must transition to the “open” conformation (22, 25–27).

### Molecular docking simulations.

To further test the idea that there is interplay between the hydroxymethyl group of MA-1-206 and the conformational flexibility of the residues comprising the hydrophobic cap, covalent docking simulations with the compound with ICM-Pro were run on a receptor model based on the OXA-23 structur: apo-OXA-23 explicitly allowing three residues (Lys82^CO2^, Val128, and Leu166) to have full side chain rotational flexibility. Prior to the simulation, the Val128 and Leu166 side chains were initially in the “closed” configuration. Following simulation, the Lys82^CO2^ side chain had moved 0.3 Å toward MA-1-206 to form a favorable hydrogen-bonding interaction with its hydroxymethyl (Fig. S5A). Additionally, both the Val128 and Leu166 side chains had transitioned to the “open” configuration; the latter was not observed in the current *in crystallo* soaking experiments with MA-1-206 but is similar to what was observed in the OXA-23-meropenem complex (22). The energy terms derived from the simulations show a more negative score (data not shown) for the poses where Leu166 has moved to the “open” conformation, suggestive of better binding to the receptor (50), and these poses may be more indicative of the actual inhibited state of the enzyme in solution.

10.1128/mbio.00367-22.10 FIG S5 Covalent docking and molecular dynamics simulations. (A). Stereoview of MA-1-206 (pink sticks) docked into a flexible receptor based on the apo-OXA-23 structure (light blue ribbons and sticks). The Lys82^CO2^, Val128, and Leu166 side chains were given full rotational flexibility during the simulation. Positions of the three side chains in the original apo-OXA-23 structure are shown as thin magenta sticks. Hydrogen bonds are shown as black dashed lines. (B). Plot of the root mean square deviation (RMSD) of the main chain (blue) and side chain atoms (orange) for the OXA-23 receptor, and for the MA-1-206 ligand (green), during the 50-ns MD simulation using Desmond. RMSDs were calculated for each 10-ps time point using the structure at *t *=* *0 as the reference. The simulation converges early and remains stable over the full 50 ns. Download FIG S5, TIF file, 0.9 MB. Copyright © 2022 Stewart et al. 2022 Stewart et al. https://creativecommons.org/licenses/by/4.0/ This content is distributed under the terms of the Creative Commons Attribution 4.0 International license.

### Molecular dynamics simulations of the MA-1-206/OXA-23 system.

**FIG 7:**
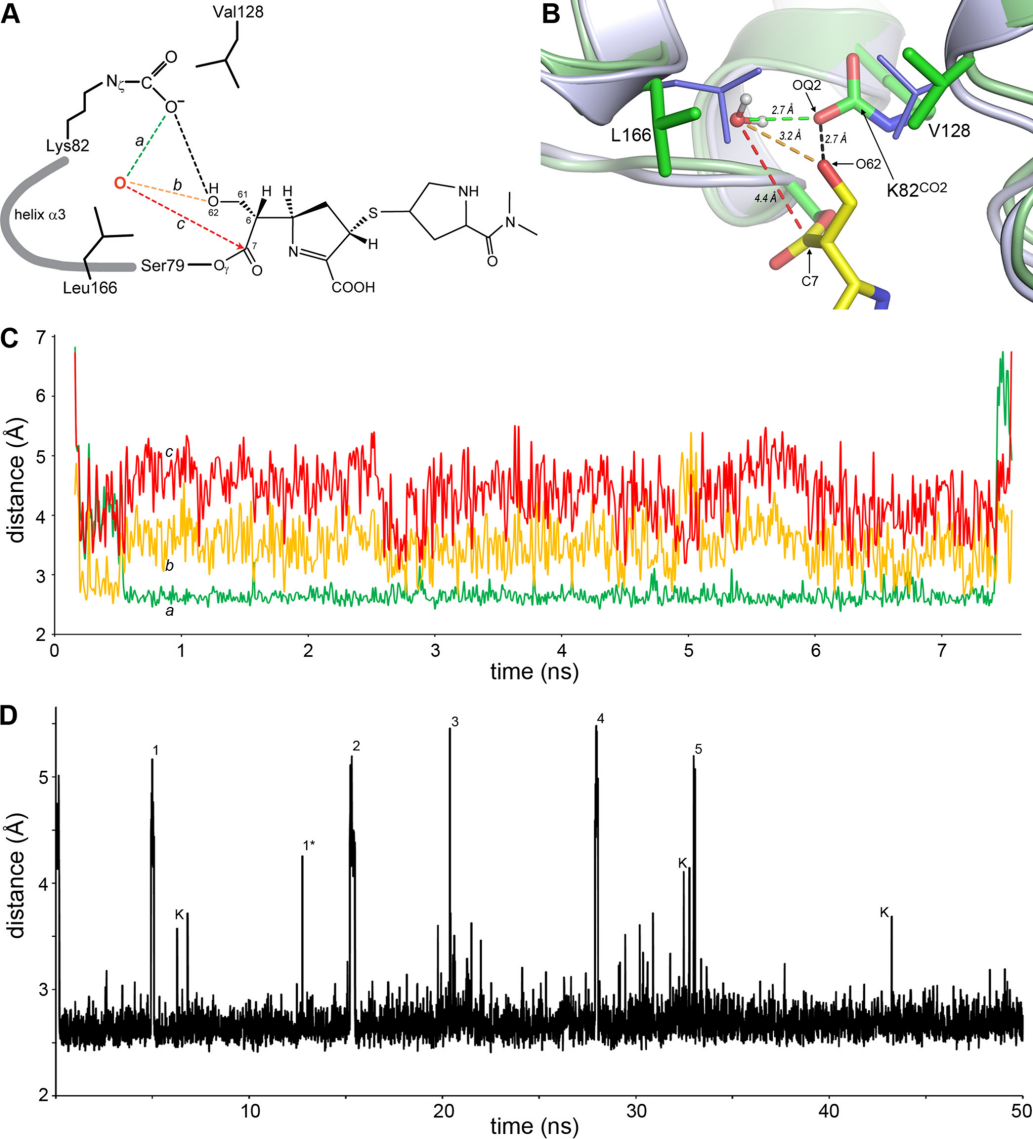
MD simulation of the OXA-23-MA-1-206 system. (A) Schematic representation of the OXA-23 active site showing the acyl enzyme intermediate formed between Ser79 and the inhibitor MA-1-206. The potential deacylating water molecule that enters the carboxylated lysine pocket is indicated as a red oxygen atom. The three distances monitored during the MD simulation for each water molecule that enters the pocket are indicated in green (*a*, to the OQ2 atom), yellow (*b*, to the O62 atom), and red (*c*, to the scissile bond represented by atom C7) dashed lines. (B) Representative frame from the 50-ns MD simulation trajectory, showing a water molecule in the carboxylated lysine pocket hydrogen bound to the OQ2 atom (green dashed line) of Lys82^CO2^ and the O62 atom (yellow dashed line) of MA-1-206 (yellow sticks). Distance to the C7 atom of MA-21-206 is indicated by the red dashed line. The Leu166 side chain is in the “open” conformation, allowing the entry of the water molecule. OXA-23 is shown as green ribbons and sticks. The positions of the Leu166 and Val128 side chains in apo-OXA-23 (light blue ribbons and thin blue sticks) are shown for comparison. The hydrogen bond between the O62 atom of MA-1-206 and the OQ2 atom of Lys82^CO2^ is shown as a black dashed line. (C) Plot of the three distances (*a*, *b*, and *c*), measured for one of the water molecules present in the carboxylated lysine pocket for approximately the first 7 ns of the MD simulation. (D) Plot of the hydrogen-bonding distance between the O62 atom of MA-1-206 and the OQ2 atom of the Lys82^CO2^ during the 50-ns MD simulation of the OXA-23/MA-1-206 system. The average distance for the interaction is 2.67 Å. The long interactions labeled 1 to 5 are instances where the hydroxymethyl group of MA-1-206 has rotated out of the carboxylated lysine pocket. The hydroxyl group remains out of the pocket for 140 ps on average (14 frames of the MD trajectory), although in the instance labeled 1*, the hydroxyl group is rotated out for only one frame. The intermediate interactions labeled K are cases where lengthening of the interaction stems from movement of the Lys82^CO2^ side chain away from the inhibitor, and these are generally for only a single frame.

To gain further insights into the interaction of MA-1-206 with OXA-23, we undertook 50-ns molecular dynamics (MD) simulations (three replicates) on a system composed of apo-OXA-23 (PDB code 4JF6) with an MA-1-206 molecule, as observed in the *t *=* *30 s complex, where the O62 hydroxyl group was initially directed away from the carboxylated lysine pocket. The root mean square deviations (RMSDs) of the protein main chain and side chain atoms and the ligand, relative to the initial model, for the duration of the 50-ns simulations indicated that the simulations were stable (Fig. S5B). Analysis of the MD trajectory showed that the O62 hydroxyl group rotates into the lysine pocket within the first 200 ps and forms a hydrogen-bonding interaction with the OQ2 atom of Lys82^CO2^ (Fig. 7A and B) with an average distance of 2.7 Å, confirming the similar observations from our structural and docking studies. We also observed movement of both the Leu166 and Val128 side chains, which comprise the hydrophobic cap, to “open” configurations; this occurred more frequently with the former (Fig. 7B), reminiscent of the “open” state observed in the OXA-23-meropenem structure (22). The opening of Leu166 was also observed in our flexible docking simulations (Fig. S5A) and suggests that when this residue is open, a channel wide enough to allow the ingress of a water molecule exists. Indeed, the MD simulations showed that opening of the hydrophobic cap by the flipping of the Leu166 and/or Val128 allows access of a water molecule from the milieu into the lysine pocket (around 15 water molecules, one at a time, entered the pocket over the course of the 50-ns simulation). The water molecule hydrogen bonds with the OQ2 atom of the carboxylated lysine and the O62 of the hydroxymethyl of MA-1-206 (Fig. 7A). Each water molecule remains in the pocket for around 5 ns on average, and once it leaves, a new water molecule enters. A representative snapshot from the MD trajectory showing one of these water molecules is shown in Fig. 7B. The average distances from the water molecule to the OQ2 and O62 atoms are 2.65 Å and 3.20 Å, respectively, suggesting that it could become activated by the carboxylated lysine. However, steric interference between the water molecule and the O62 of the hydroxymethyl group restricts its approach to the C7 atom (representing the atom where an activated deacylating water would attack) to approximately 4.4 Å (Fig. 7B and C), which would prevent deacylation. The only scenario in which deacylation could occur would be if the hydroxymethyl group were to move away from its current position, thus resolving the steric interference. Indeed, breakage of the hydrogen bond between the O62 atom of the hydroxymethyl with the OQ2 atom of the carboxylated lysine and subsequent rotation of the hydroxymethyl group out of the lysine pocket was observed at least five times over the course of the MD simulations (Fig. 7D). The results described above were consistent in the three replicate simulations.

Combined, our kinetic, structural, molecular docking, and MD simulation data allowed us to evaluate the interaction between OXA-23 and MA-1-206 and gain insights into the mechanism of enzyme inhibition. Kinetic experiments revealed dramatic differences in the reaction of MA-1-206 versus the commercial carbapenems meropenem and imipenem with OXA-23. We observed that during the initial fast phase of the reaction, MA-1-206 was hydrolyzed at almost the same rate as meropenem and at a slower rate than imipenem; however, this rate dramatically decreased after the first 11 turnovers of MA-1-206, leading to a much slower phase of the reaction where the residence time of the compound was nearly 40 min. Thus, MA-1-206 is a reversible inhibitor of OXA-23, while meropenem and imipenem are substrates of the enzyme. Structural studies showed that all three antibiotics adopted the Δ^1^*S* tautomeric conformation upon forming covalent intermediates with OXA-23, which strongly indicates that the Δ^1^*S* tautomeric conformation is not a significant contributor to the observed differences in kinetics. In contrast, the generated data show that the C6 hydroxymethyl substituent of MA-1-206 is responsible for the drastic attenuation of hydrolysis. Previously, it was demonstrated that hydrolysis of carbapenems by CHDLs would require opening of the hydrophobic cap to allow access of the deacylating water to the active site, which could be achieved by movement of Val128, Leu166, or both simultaneously to the “open” conformation (22, 25, 26). Upon this opening, the canonical hydroxyethyl group of carbapenems is restricted from movement (22, 25). However, as we show here, movement of either of these residues would allow the smaller hydroxymethyl group of MA-1-206 to freely rotate and test several available rotomeric conformations; lack of electron density for the hydroxymethyl group for at least the first 2 min indicates that it is indeed highly mobile in the majority of MA-1-206 molecules. In one of these rotomeric conformations, the group is positioned in close proximity to the carboxylated lysine of OXA-23, allowing the formation of a hydrogen bond. Our MD simulations showed that upon formation of the hydrogen bond, the hydroxymethyl group sterically prevented access of the deacylating water to the scissile bond, converting the complex into the inactive form. The number of such molecules would progressively increase over time, resulting in inhibition. Our MD simulations also demonstrated that the inactive species reverts back to the reactive species as a result of breakage of the hydrogen bond and outward rotation of the hydroxymethyl group, allowing the deacylating water to access the scissile bond. However, this process proceeds with low efficiency, as evident from the slow rate of reactivation observed following inhibition of OXA-23. These data allow us to evaluate the validities of the different proposed kinetic mechanisms (Fig. 3). Per Fig. 3B, the onset of reversible inhibition of OXA-23 occurs after formation of the noncovalent Michaelis complex (*ES*). However, our structural information argues against this pathway, as we did not observe accumulation of such a complex upon soaking of OXA-23 crystals with MA-1-206. According to Fig. 3C and D, the onset of reversible inhibition of OXA-23 occurs after formation of the covalent acyl-enzyme complex (*E-S*), which is supported by our structural data. However, our MD simulations showed that the *E-I* complex cannot deacylate without reverting back to the *E-S* species, which is in full agreement with the pathway shown in Fig. 3D. The studies presented here validate the strategy of retooling existing carbapenems to restore their efficacy in fighting deadly MDR*Ab* infections.

## MATERIALS AND METHODS

### Strains and plasmids.

Cloning of the OXA genes under the ISAba3 promoter in the pNT221 Acinetobacter baumannii*-*Escherichia coli shuttle vector designed in our lab has been previously described (22, 51). The gene encoding wild-type ADC-1 from A. baumannii with its own leader sequence (GenBank accession no. AJ009979) was custom-synthesized (GenScript) and cloned between NdeI and HindIII restriction sites in the pNT255 A. baumannii*-*E. coli shuttle vector designed in our lab, which contains the ISAaba1 promoter. The genes for wild-type KPC-6 (GenBank accession no. TAH98735.1) and GES-5 (GenBank accession no. WP_012658785.1), with the OmpA leader sequence and wild-type VIM-2 (GenBank accession no. NC_020452.1) and IMP-1 (GenBank accession no. S71932.1), with their own leader sequences were custom-synthesized (Synbio Technologies) and cloned between NdeI and HindIII restriction sites in pNT221. The gene encoding wild-type TEM-1 with the OmpA leader was cloned between NdeI and HindIII restriction sites in pNT221. For some of the genes, NdeI or HindIII restriction sites were present and were removed by introducing silent mutations. The clinical isolates used for MIC testing were obtained from the Acinetobacter baumannii and Gram-negative Carbapenemase Detection Panels from the CDC & FDA Antibiotic Resistance (AR) Isolate Bank (52).

### Protein expression and purification.

For kinetic, liquid chromatography/mass spectrometry, and crystallographic studies, OXA-23 was expressed and purified as previously described (22). To measure the extinction coefficient of MA-1-206, KPC-6 was expressed and purified as previously described (53).

### Antimicrobial susceptibility testing.

MICs of MA-1-206, meropenem, and imipenem were measured against A. baumannii CIP 70.10 producing various β-lactamases and against A. baumannii clinical isolates from the CDC & FDA AR Isolate Bank according to CLSI guidelines (54). Briefly, bacteria were inoculated at a final concentration of 5 × 10^5^ CFU/mL into a 96-well plate containing a series of 2-fold dilutions of the antibiotics in Mueller-Hinton II broth (Difco). The plates were incubated at 37°C for 20 to 24 h prior to interpretation of the results. All measurements were performed in triplicate.

### Enzyme kinetics.

All data were collected in at least triplicate at 22°C using either a Cary 60 spectrophotometer (Agilent) or an SFM-300 stopped-flow instrument (Bio-Logic) and analyzed using nonlinear regression in Prism 5 (GraphPad Software, Inc.). All reactions were performed in 100 mM sodium phosphate (pH 7).

**Measurement of the molar extinction coefficient of MA-1-206.** Absorbances of various concentrations (2 to 200 μM) of MA-1-206 before and after hydrolysis by KPC-6 (70 nM) were measured at 298 nm. The change in absorbance was plotted versus the concentration of MA-1-206, and the molar extinction coefficient (Δε = −6,080 ± 60 M^−1^cm^−1^) was obtained from the slope of the line.

**Inhibition of OXA-23 by MA-1-206.** Reaction mixtures containing 20 to 100 μM MA-1-206, 0.5 to 1 μM OXA-23, 0.2 mg/mL bovine serum albumin (BSA), and 50 mM NaHCO_3_ were monitored at 298 nm for 30 to 60 min. The number of turnovers prior to inhibition of OXA-23 was determined from the molar ratio of MA-1-206 to enzyme which showed >98% reduction of enzyme activity. Control reactions were performed under the same conditions in the absence of either the compound or OXA-23.

**Determination of the partition ratio.** The partition ratio was determined using the titration method (55). Reaction mixtures containing varying molar ratios (up to 100) of MA-1-206 to OXA-23, 0.2 mg/mL BSA, and 50 mM NaHCO_3_ were incubated for 45 min and subsequently diluted 200- to 500-fold into 400 μM nitrocefin (λ = 500 nm, Δε = +15,900 M^−1^cm^−1^) containing 0.2 mg/mL BSA and 50 mM NaHCO_3_. The final enzyme concentration was 100 to 200 pM. Control reactions were performed under the same conditions, but in the absence of MA-1-206. The absorbance was monitored at 500 nm, and fractional activity was determined from the initial velocities in the presence and absence of the compound. The remaining enzyme activity was plotted against the molar ratio of MA-1-206 to enzyme, and the partition ratio was calculated as previously described (56). Because the higher-ratio data points deviated from linearity, only the linear portion of the curve was used for extrapolation.

**Determination of the dissociation constant, K_i_, and second-order rate constant for inactivation, *k*_inact_/*K*_I_.** The dissociation constant, *K*_i_ (*K*_i_ = *k*_-1_/*k*_1_), was determined in a competition experiment using nitrocefin as a reporter substrate. Reaction mixtures containing 0 to 1 μM MA-1-206, 50 mM NaHCO_3_, 0.2 mg/mL BSA, and either 200 or 400 μM nitrocefin were initiated by the addition of 200 pM OXA-23. The absorbance was monitored at 500 nm and the progress curves were fit to Equation 1:

(1) $$A_{\text{t}}=A_{0}\;+\;v_{\text{S}}t\;+\;\frac{v_{\text{i}}\;-\;v_{\text{S}}}{k_{\text{inter}}}(1\;-\;e^{-k_{\text{inter}}t})$$

where *A*_t_ is the absorbance at time *t*, *A*_0_ is the initial absorbance, *v*_i_ is the initial velocity, *v*_s_ is the steady-state velocity, and *k*_inter_ is the first-order rate constant for the interconversion between *v*_i_ and *v*_s_. Initial velocities were plotted against the concentration of MA-1-206, and data were fit to the Morrison equation (Equations 2 and 3) (33):

(2) v=v0(1−E+I+Kiapp−(E+I+Kiapp)2−4EI2E)

(3) $$K_{\text{i}}^{\text{app}}=K_{\text{i}}\left(1\;+\;\frac{S}{K_{m}}\right)$$

where *K*_i_ is as described above, *E* is the concentration of OXA-23, *I* is the concentration of MA-1-206, *v* is the velocity in the presence of inhibitor, *v*_0_ is the velocity in the absence of inhibitor, *S* is the concentration of nitrocefin, and *K*_m_ is the Michaelis constant for nitrocefin. To determine the second-order rate constant for inactivation, *k*_inact_/*K*_I_, *k*_inter_ values obtained in the presence of 200 μM nitrocefin were plotted against the MA-1-206 concentration and the data were fit to Equation 4:

(4) $$k_{\text{inter}}=\frac{k_{\text{inact}}(I)}{K_{\text{I}}\left(1\;+\;\frac{S}{K_{m}}\right)\;+\;I}$$

where *k*_inact_ is the rate constant for inactivation and *K*_I_ is the concentration of inhibitor required to reach 1/2 *k*_inact_.

**Determination of the acylation rate constant, *k*_2_.** Single-turnover conditions were utilized to measure acylation of OXA-23 by MA-1-206, meropenem (λ = 298 nm, Δε = −7,200 M^−1^cm^−1^), and imipenem (λ = 297 nm, Δε = −10,930 M^−1^cm^−1^). Reaction mixtures containing 10 μM carbapenem were initiated by addition of increasing concentrations of OXA-23 (50 to 200 μM). The data for acylation of MA-1-206 were fit to Equation 5

(5) $$A_{\text{t}}=(A_{0}\;-\;A_{\infty }){\text{e}}^{-k_{2}t}\;+\;A_{\infty }$$

where *A*_t_, *A*_0_, and *t* are as described above, *A*_∞_ is the final absorbance, and *k*_2_ is the observed first-order rate constant for acylation. The data for acylation of meropenem and imipenem were fit to Equation 6:

(6) $$A_{\text{t}}=(A_{0}\;-\;A_{\infty })(F_{\text{fast}}){\text{e}}^{-k_{\text{2fast}}t}\;+\;(A_{0}\;-\;A_{\infty })(1\;-\;F_{\text{fast}}){\text{e}}^{-k_{\text{2slow}}t}\;+\;A_{\infty }$$

where *A*_t_, *A*_0_, *A*_∞_, and *t* are as described above, *F*_fast_ is the fraction of the reaction corresponding to the fast phase, *k*_2 fast_ is the observed first-order rate constant for the fast phase of acylation, and *k*_2 slow_ is the observed first-order rate constant for the slow phase of acylation.

**Determination of the deacylation and reactivation rate constants, *k*_3_ and *k*_r_.** The jump dilution method (57) was used to measure the recovery of activity of OXA-23 after incubation with either MA-1-206 or meropenem. For meropenem, reaction mixtures containing 200 nM OXA-23, 5 μM meropenem, 0.2 mg/mL BSA, and 50 mM NaHCO_3_ were incubated for 30 sec and subsequently diluted 1,000-fold into 400 μM nitrocefin containing 0.2 mg/mL BSA and 50 mM NaHCO_3_. Because MA-1-206 progressively inhibits OXA-23 over a period of 15 to 20 min, to measure the rate of recovery from inhibition (*k*_r_ in Fig. 3B–D), we incubated reaction mixtures containing 20 nM OXA-23, 500 nM MA-1-206, 0.2 mg/mL BSA, and 50 mM NaHCO_3_ for 30 min (to ensure full inhibition). Subsequently, the reactions were diluted 2,000-fold into 400 μM nitrocefin containing 0.2 mg/mL BSA and 50 mM NaHCO_3_. Incubation times (up to 30 sec for meropenem and 10 to 30 min for MA-1-206) and dilution ratios (100- to 2,000-fold) were varied to ensure full inhibition and subsequent maximal recovery of enzyme activity, respectively. Control reactions were performed under the same conditions, but in the absence of MA-1-206 or meropenem. The absorbance was monitored at 500 nm, and the progress curves were fit to Equation 7:

(7) $$A_{t}=A_{0}\;+\;v_{\text{S}}t\;-\;\frac{v_{\text{S}}}{k}\text{(1}\;-\;{\text{e}}^{-\mathrm{kt}})$$

where *A*_t_, *t*, *A*_0_, and *v*_s_ are as described above and *k* represents either the rate constant for deacylation, *k*_3_, or the rate constant for reactivation, *k*_r_. Because the deacylation rate for imipenem was too fast to measure experimentally, *k*_3_ was calculated using Equation 8:

(8) $$k_{\text{cat}}=\frac{k_{2}k_{3}}{k_{2}\;+\;k_{3}}$$

where *k*_2_ and *k*_3_ are as described above and *k*_cat_ is the turnover number.

### Liquid chromatography/mass spectrometry (LC/MS) experiments.

To analyze products from the fast phase of the reaction, MA-1-206 (20 μM) and OXA-23 (5 μM) in 100 mM sodium phosphate (pH 7) supplemented with 50 mM NaHCO_3_ were incubated at 22°C. After 5 and 10 min, the solution was passed through an Amicon Ultra-0.5 mL centrifugal filter (10-kDa molecular weight cut-off [MWCO]) (Millipore) for 5 min at 14,000 × *g* to remove the enzyme and analyzed using LC/MS as described below. A control was performed under the same conditions, but in the absence of the enzyme. To analyze products from the slow phase of the reaction, MA-1-206 (600 μM) and OXA-23 (40 μM) in 100 mM sodium phosphate (pH 7) supplemented with 50 mM NaHCO_3_ were incubated for 1 h at 22°C to allow for full inhibition of the enzyme. Subsequently, excess compound was removed by passing the reaction through three successive Zeba-0.5 mL spin desalting columns (7-kDa MWCO) (Thermo Fisher) according to the manufacturer’s instructions. After the reaction mixture was incubated for an additional 3 h at 22°C to allow for product formation, it was passed through an Amicon Ultra-0.5 mL centrifugal filter (10-kDa MWCO) (Millipore) for 12 min at 14,000 × *g* to remove the enzyme and analyzed using LC/MS as described below. For LC/MS analysis, the instrument consisted of an ultra-high pressure LC system coupled with a Bruker micrOTOF-QII mass spectrometer using Hystar 5.0 SR1 software. The electrospray ionization source was run in positive-ion mode using the following parameters: end plate offset voltage = −500 V, capillary voltage = 2,000 V, and nitrogen as both a nebulizer (4 bar) and dry gas (7 L/min) at 180°C. Mass spectra were collected from 50 to 3,000 *m/z*. LC separations were accomplished using a Waters Acquity UPLC HSS T3 C-column (1.8 μm, 2 × 150 mm) at 40°C with a 15-min gradient (2-min hold at 98% A/2% B, followed by a 8.9-min linear gradient to 15% B, a 0.1-min linear gradient to 98% A/2% B, and then a 4-min hold; A = 0.1% formic acid in water, B = 0.1% formic acid in acetonitrile) at a flow rate of 0.4 mL/min. For the first 2 min of each run, LC flow was diverted to the waste.

### MA-1-206 and imipenem soaking experiments.

Crystals of apo-OXA-23 at neutral pH were grown using conditions previously described (22). Briefly, the enzyme was crystallized by sitting drop in 96-well Intelliplates (Art Robbins), using 0.2 M succinic acid and 20% PEG3350 (pH 7.0) as the reservoir solution. The crystals grew overnight to approximately 75 to 150 μm. Crystals of approximately equal sizes were used for soaking experiments. Binding of MA-1-206 was initiated by transferring apo-OXA-23 crystals into crystallization buffer augmented with 25% ethylene glycol as a cryoprotectant and containing 50 mM MA-1-206. At prescribed time points (Table S1), the crystals were removed from the soaking buffer and flash-cooled in liquid nitrogen to quench the acylation reaction. The soaked crystals diffracted to between 2.3 and 2.65 Å resolution. For the imipenem-soaking experiments, apo-OXA-23 crystals were transferred into crystallization buffer augmented with 25% ethylene glycol as a cryoprotectant and containing 50 mM imipenem for 2 min prior to flash-cooling in liquid nitrogen. The crystals diffracted to approximately 2.4 Å resolution.

### Data collection, structure solution, and refinement.

Complete data sets were collected from the MA-1-206-soaked crystals using SSRL beamline BL12-2 using a Dectris PILATUS 6M PAD detector running in shutterless mode and X-rays at 12,658 eV (0.97946 Å). The images were processed with XDS (58) and scaled and merged using AIMLESS (59). The structures were solved by molecular substitution with MOLREP (60) using apo-OXA-23 at pH 7.0 (PDB code 4JF6) as the starting model with all water molecules removed. The structures were initially refined using a single round REFMAC (61), and difference-density maps were calculated from the resulting model and inspected for the presence of MA-1-206 in the active site. Interactive model building with COOT (62) was used subsequently to add water molecules and MA-1-206. Refinement of the seven MA-1-206 complexes was completed with *phenix.refine* (63). Data collection statistics for the various soaking time points are given in Table S2 and refinement statistics in Table S3.

10.1128/mbio.00367-22.3 TABLE S2 OXA-23-MA-1-206 soak data collection statistics. Download Table S2, DOCX file, 0.01 MB. Copyright © 2022 Stewart et al. 2022 Stewart et al. https://creativecommons.org/licenses/by/4.0/ This content is distributed under the terms of the Creative Commons Attribution 4.0 International license.

10.1128/mbio.00367-22.4 TABLE S3 OXA-23-MA-1-206 soak refinement statistics. Download Table S3, DOCX file, 0.01 MB. Copyright © 2022 Stewart et al. 2022 Stewart et al. https://creativecommons.org/licenses/by/4.0/ This content is distributed under the terms of the Creative Commons Attribution 4.0 International license.

A complete data set was collected from the imipenem-soaked OXA-23 crystal via SSRL Beamline BL9-2. The images were processed with XDS (58) and scaled and merged using AIMLESS (59). The structure was solved by molecular substitution using apo-OXA-23 at pH 7.0 (PDB code 4JF6) as the starting model with all water molecules removed. The structure was initially refined using REFMAC (61), and difference-density maps were calculated from the resulting model. Refinement was completed with *phenix.refine* (63). Data collection and refinement statistics are given in Table S4.

10.1128/mbio.00367-22.5 TABLE S4 OXA-23-imipenem soak data collection and refinement statistics. Download Table S4, DOCX file, 0.01 MB. Copyright © 2022 Stewart et al. 2022 Stewart et al. https://creativecommons.org/licenses/by/4.0/ This content is distributed under the terms of the Creative Commons Attribution 4.0 International license.

### Computational methods.

Figures were generated with PyMOL (64). Solvent accessible surfaces were also calculated with PyMOL, using a probe radius of 1.4 Å (equivalent to the radius of a single water molecule). Covalent docking of MA-1-206 to apo-OXA-23 was carried out with ICM-Pro 3.9-1c (Molsoft) (65, 66). Apo-OXA-23 (PDB code 4JF6) was converted to an ICM object with optimization of hydrogen atom placement. The OXA-23-meropenem structure (PDB code 4JF4) was superimposed and used to define an initial position for the substrate-binding site in the apo-OXA-23 receptor. MA-1-206 was docked to the receptor using the ICM-Pro covalent-docking procedure. Additional covalent-docking simulations were performed using flexible receptor procedures, allowing the side chains of Lys82^CO2^, Val128, and Leu166 to be fully rotatable. The MA-1-206 docking runs were performed multiple times, and the most energetically favored binding modes were extracted from ICM-Pro as PDB files.

Molecular dynamics simulations were performed in triplicate on a composite model comprising apo-OXA-23 (PDB code 4JF6) with an MA-1-206 acyl intermediate derived from the *t *=* *30 s complex, using Desmond (67) in the Schrodinger 2019-2 release. The composite complex was prepared with Maestro (Schrodinger) using the OPLS3e force field (68). The predefined TIP3P water model (69) was used to build the system. The overall charge of the complex was calculated as +2 and neutralized with two Cl^−^ ions, and 0.15 M salt (NaCl) was added prior to building the system. The system was minimized prior to the final 50-ns production step run at 300 K and 1 Atm pressure, using the Nosé-Hoover chain coupling scheme for temperature control and the Martyna-Tuckerman-Klein chain coupling scheme with a coupling constant of 2.0 ps for pressure control (70). Non-bonded forces were calculated using an r-RESPA integrator. The trajectories were saved at 10-ps intervals for analysis. Maestro and Desmond were run on the SHERLOCK 3.0 HPC cluster at Stanford University.
