# Supplementary figures and images for: C6 Hydroxymethyl-Substituted Carbapenem MA-1-206 Inhibits the Major Acinetobacter baumannii Carbapenemase OXA-23 by Impeding Deacylation
Source: mBio. 2022 Apr 14;13(3):e00367-22. doi: 10.1128/mbio.00367-22 (PMC9239083; doi:10.1128/mbio.00367-22)

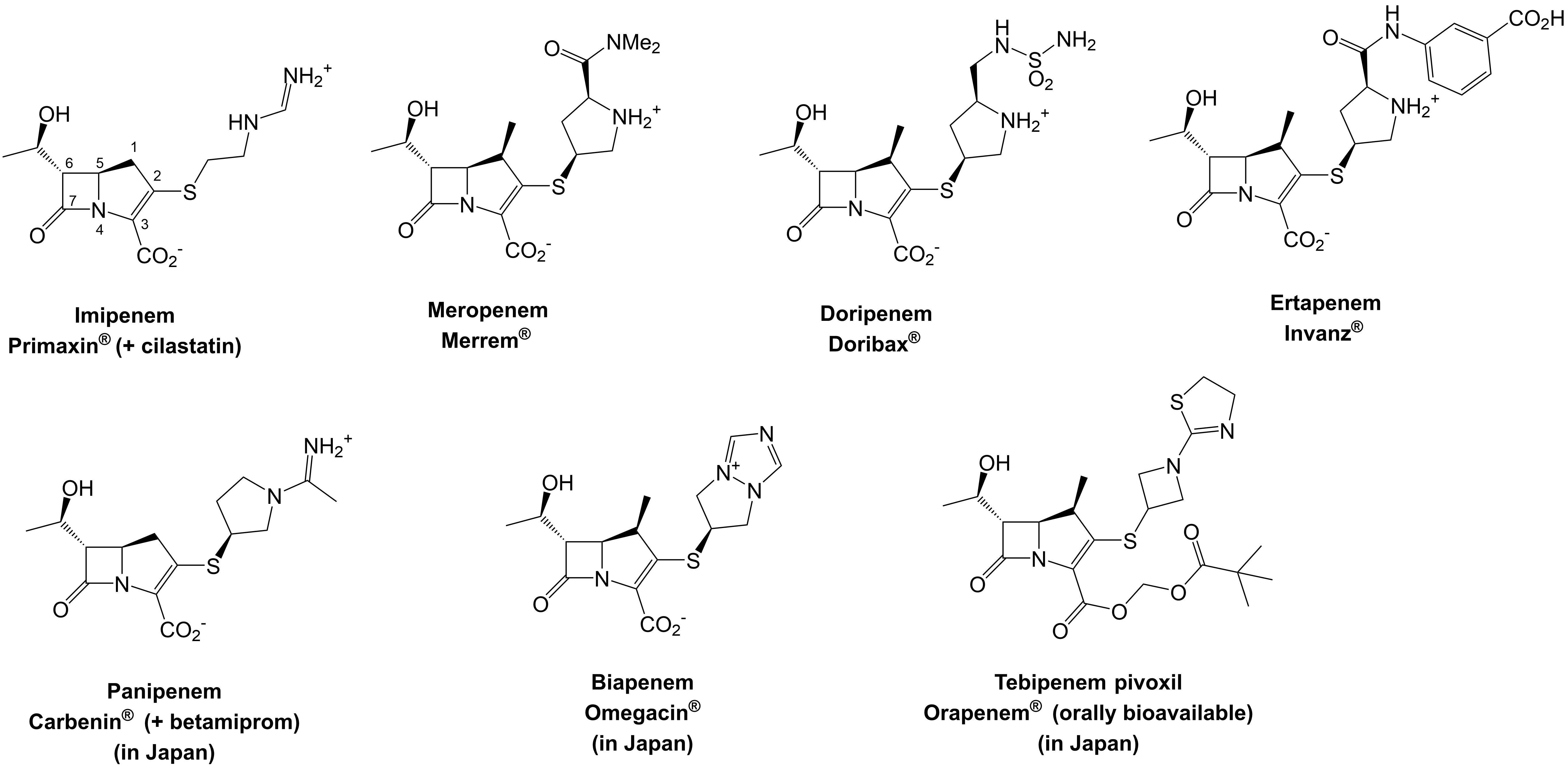

Supplement: FIG S1 [file mbio.00367-22-s0002.tif]

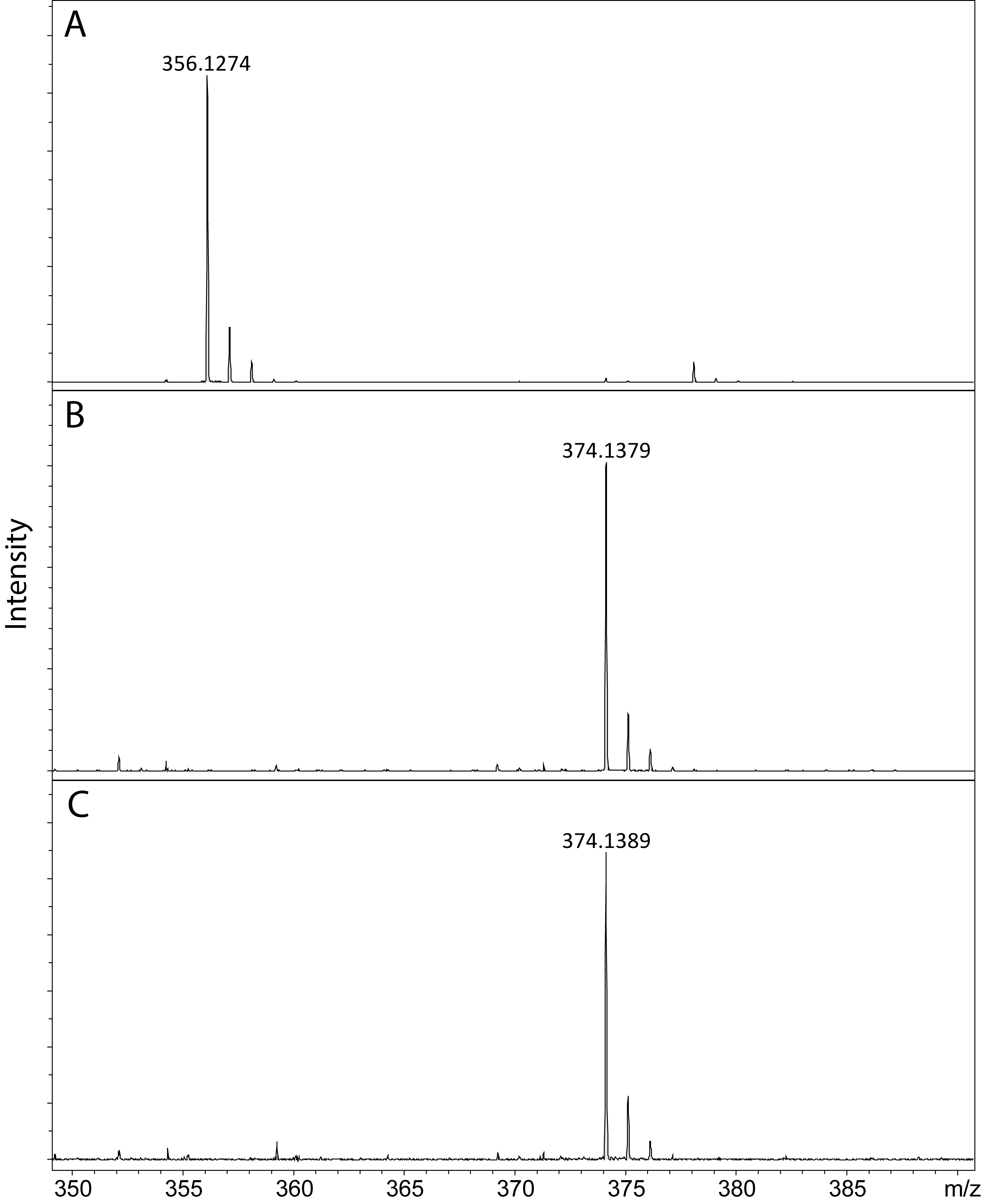

Supplement: FIG S2 [file mbio.00367-22-s0003.tif]

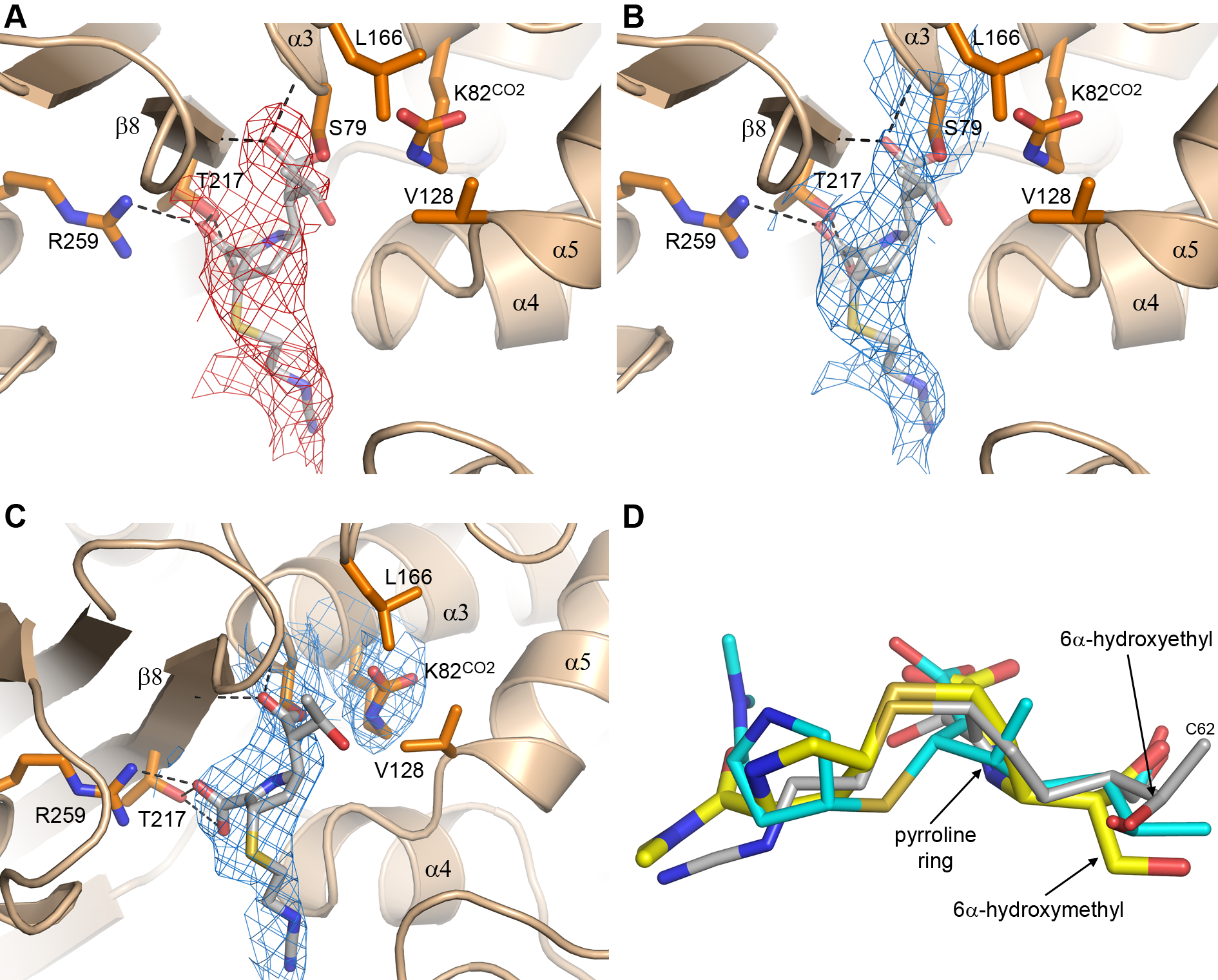

Supplement: FIG S3 [file mbio.00367-22-s0004.tif]

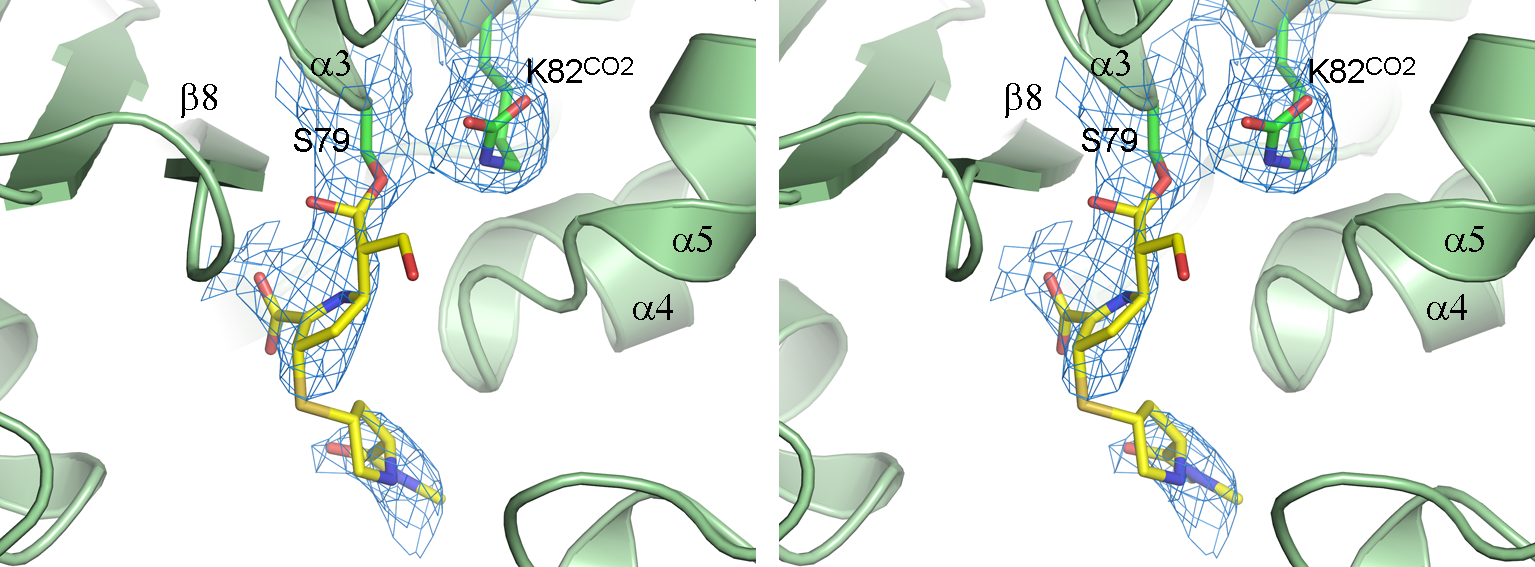

Supplement: FIG S4 [file mbio.00367-22-s0005.tif]

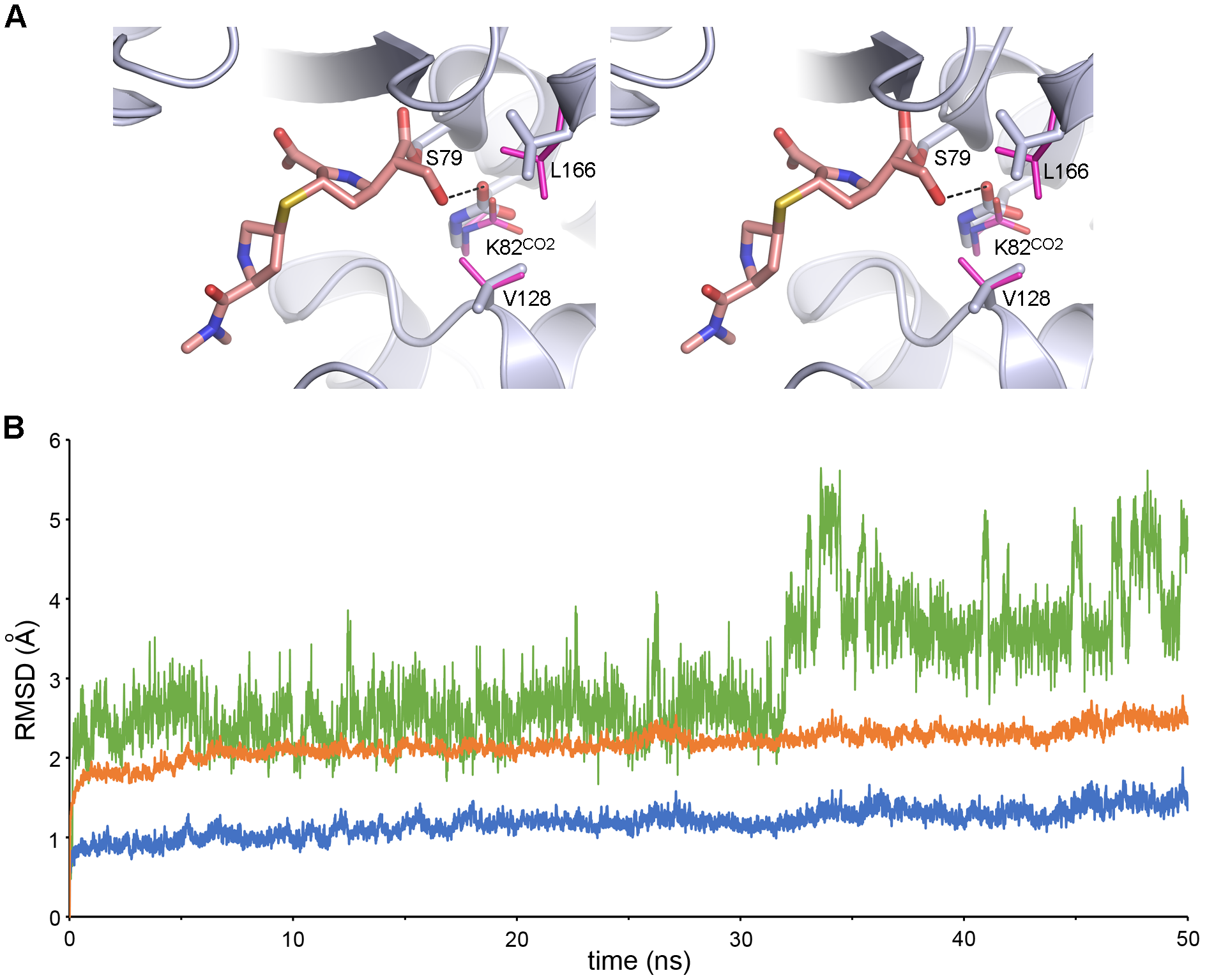

Supplement: FIG S5 [file mbio.00367-22-s0006.tif]
