# Supplementary material for: C6 Hydroxymethyl-Substituted Carbapenem MA-1-206 Inhibits the Major Acinetobacter baumannii Carbapenemase OXA-23 by Impeding Deacylation
Source: mBio. 2022 Apr 14;13(3):e00367-22. doi: 10.1128/mbio.00367-22 (PMC9239083; doi:10.1128/mbio.00367-22)
Supplement: TEXT S1 [file mbio.00367-22-s0001.docx]

**Text S1. Procedures for synthesis of MA-1-206**

(p-Nitrophenyl)methyl 4-[3-(1-*tert*-butyldimethysilyoxyethyl)-4-oxo-2-azetidinyl]-2-diazoacetoacetate **3.**

To a solution of commercially available acetate **1** (389 g, 1.49 mole) and TBS enol ether **2** (562 g, 1.49 mole) in dry CH_2_Cl_2_, was added solid ZnCl_2_ at room temperature. The reaction was briefly warmed to 40 ^o^C and then allowed to stir without external heating for 3 h. The solution was then washed with saturated sodium bicarbonate (2 X) and then brine, dried (Na_2_SO_4_) and evaporated, yielding a crude oily yellow solid. After purification by column chromatography (EtOAc/DCM) the yield was (392 g, 53%). ^1^H NMR (400 MHz, CDCl_3_): δ 8.27 (d, 2H), 7.55 (d, 2H), 6.15 (s, 1H), 5.38 (s, 2H), 4.08 (td, 1H), 3.67 (d, 1H), 3.48 (dd, 1H), 2.86 (dd, 1H), 2.07 (d, 3H), 0.86 (s, 9H), 0.04 (d, 6H). IR (KBr, cm^−1^): 3360, 2955, 2930, 2885, 2857, 2142, 1721, 1655, 1608, 1524, 1385, 1348, 1295, 1257, 1215, 1141, 1052, 1014, 837, 778, 74. HRMS (ESI-TOF): *m/z* calcd for C_22_H_30_N_4_O_7_Si 513.1776, found 513.1765 [M+Na]^+^.

(p-Nitrophenyl)methyl 4-[3-(1-hydroxyethyl)-4-oxo-2-azetidinyl]-2-(diazo)acetoacetate **4**.

30 mL of 48% aq HF was added to a solution of compound **3** (392 g, 776 mmol) in 600 mL of CH_3_CN and the reaction allowed to stir at rt overnight. The reaction was monitored by thin layer chromatography. After stirring overnight, an additional 20 mL of 48% aq HF was added to the reaction to complete. After the reaction completed, fine powder of NaHCO_3_ was added to the mixture to adjust pH to 8. The solid was filtered and the filtrate was concentrated under reduced pressure to afford a white solid (296 g, 98 %). ^1^H NMR (400 MHz, CDCl_3_): δ 8.28 (d, 2H), 7.56 (d, 2H), 6.27 (s, 1H), 5.38 (d, 2H), 4.40 (m, 1H), 3.97 (d, 2H), 3.97 (s, 1H), 3.42 (dd, 1H), 3.09 (dd, 1H), 2.34 (s, 3H). ^13^C NMR (CDCl_3_, 100 MHz): δ 190, 161, 148, 142, 129, 124, 65.6, 65.1. IR (KBr, cm^−1^): 3390, 2967, 2142, 1721, 1651, 1608, 1522, 1386, 1347, 1295, 1217, 1129, 1015, 853, 739. HRMS (ESI-TOF): *m/z* calcd for C_16_H_16_N_4_O_7_ 399.0911, found 399.0884 [M+Na]^+^.

(p-Nitrophenyl)methyl 4-(3-acetyl-4-oxo-2-azetidinyl)-2-(imino)acetoacetate **5**

Compound **4** (20.0 g, 53.1 mmol) was dissolved in dry DCM (200 mL). Dess-Martin periodinane (22.5 g, 55.2 mmol) was added slowly over 20 min. The reaction stirred for 15 min at rt. A solution of sodium thiosulfate pentahydrate (20 g) in a saturated solution of NaHCO_3_ (300 mL) was added to the reaction and stirred for an additional h until DMP disappeared in NMR spectra. The organic layer was separated and washed with water. Then, the organic layer was concentrated under reduced pressure. The yield was (19 g, 95%). ^1^H NMR (400 MHz, CDCl_3_): δ 8.27 (d, 2H), 7.55 (d, 2H), 6.27 (s, 1H), 5.38 (s, 2H), 4.39 (m, 1H), 3.96 (d, 1H), 3.42 (dd, 1H), 3.11 (dd, 1H), 2.35 (s, 3H). ^13^C NMR (CDCl_3_, 100 MHz): δ 189, 163, 161, 142, 129, 124, 68.4, 65.6, 53.4, 45.6, 44.2, 29.7. IR (KBr, cm^−1^): 3287, 2146, 1763, 1709, 1644, 1523, 1384, 1348, 1295, 1212, 1119, 1014, 853, 696, 718. HRMS (ESI-TOF): *m/z* calcd for C_16_H_14_N_4_O_7_ 397.0755, found 397.0729 [M+Na]^+^.

(p-Nitrophenyl)methyl 4-[3-(1-tertbutyldimethysilyoxyethenyl)-4-oxo-2-azetidinyl]-2-(imino)acetoacetate **6**

Compound **5** (18.8g, 38.5 mmol) was dissolved in CH_2_Cl_2_ (120 mL) then hexanes (80 mL) were added. The compound was allowed to dissolve and stirred at rt for 5 min. Then the reaction was cooled to -20 $℃$. DIPEA (26.8 mL, 154 mmol) was added followed by TBS-OTf (35.4 mL, 154 mmol). The reaction was stirred for 40 min at rt. After completion, the resulting mixture was diluted with CH_2_Cl_2_ (50 mL), then washed with NaHCO_3_ and water. Then the organic phase was separated and dried over Na_2_SO_4_, and concentrated under reduced pressure. The title compound purified by column chromatography eluted (0-10%) EtOAc/DCM to obtain (15.7 g, 61%) of the title compound. ^1^H NMR (400 MHz, CDCl_3_): δ 8.28 (d, 2H), 7.55 (d, 2H), 5.38 (s, 2H), 4.18 (d, 2H), 3.91 (d, 1H), 3.50 (m, 1H), 2.80 (d, 1H), 2.05 (s, 3H), 1.98 (s, 3H), 1.70 (s, 3H), 0.87 (s, 3H), 0.06 (s, 12H). ^13^C NMR (100 MHz, CDCl_3_): δ 189, 171, 161, 152, 148, 141.9, 129, 123, 92.5, 65.4, 63.9, 49.9, 46.1, 26.2, 25.7, 25.6, 25.4, 18.2, 18.1, -3.00, -3.63, -4.53, -4.74, -5.41, -5.99. IR (KBr, cm^−1^): 2956, 2931, 2858, 2140, 1746, 1721, 1656, 1525, 1348, 1312, 1255, 840. HRMS (ESI-TOF): *m/z* calcd for C_28_H_42_N_4_O_7_Si_2_ 625.2484, found 625.2465 [M+Na]^+^.

4-{3-(Imino)-3-[(p-nitrophenyl)methoxycarbonyl]-2-oxopropyl}-2-oxo-3-azetidinecarboxylic acid **7**

A solution of the silyl enol ether **6** (7.30 g, 12.4 mmol) in DCM (100 mL) was treated with O_3_ at -78 °C until a blue color persisted. The solution was then purged with a stream of N_2_ bubbles until it was colorless. To the resulting solution, Me_2_S (10 mL) was added, and the mixture was stirred at rt for 2 h and washed with cold water twice. The organic layer was separated and dried over Na_2_SO_4_ and concentrated to afford a viscous solid (6.1 g, 81%). ^1^H NMR (400 MHz, CDCl_3_): δ 8.29 (d, 2H), 7.57 (d, 2H), 5.39 (s, 2H), 4.21 (dd, 1H), 3.90 (d, 1H), 3.70 (d, 1H), 2.98 (m, 1H), 0.96 (m, 18H), 0.04 (m, 12H). ^13^C NMR (100 MHz, CDCl_3_): δ 173, 168, 167, 153, 138, 129, 129, 128.9, 127, 126, 125, 69.8, 60.5, 57.7, 55.3, 38.3, 26.6, 26.1, 25.9, 25.6, 25.5, 25.4, 18.9, 17.8, 15.4, 15.1, -3.63, -4.75, -4.92, -5.16, -5.87, -5.96. IR (KBr, cm^−1^): 2957, 2931, 2860, 2349, 2142, 1754, 1720, 1655, 1608, 1525, 1472, 1381, 1348, 1315, 1257, 1196, 1129, 1001, 948, 842, 809, 742, 696. HRMS (ESI-TOF): *m/z* calcd for C_27_H_40_N_4_O_8_Si_2_ 605.2457, found 605.2479 [M+H]^+^.

(p-Nitrophenyl)methyl 4-[3-(chloroformyl)-4-oxo-2-azetidinyl]-2-(imino)acetoacetate **8**

A solution of the TBS ester **7** (6.3 g, 10.4 mmol) in DCM (50 mL) was cooled at 0 °C under N_2_, and DMF (5 drops) was added, followed by oxalyl chloride (3.57 mL, 41.6 mmol). The mixture was stirred at rt for 0.5 h. The resulting yellow solution was evaporated to dryness to afford the titled compound **34** (5.29 g, 99%). ^1^H NMR (400 MHz, CDCl_3_): δ 8.29 (d, 2H), 7.57 (d, 2H), 5.38 (s, 2H), 4.26 (d, 2H), 4.25 (m, 1H), 3.65 (dd, 1H), 2.98 (dd, 1H), 0.88 (s, 9H), 0.05 (d, 6H). ^13^C NMR (100 MHz, CDCl_3_): δ 188, 167, 163, 160, 148, 142, 129, 142, 129, 124, 71.6, 65.6, 49.6, 44.6, 25.9, 18.4, 0.96, -5.57, -5.87. IR (KBr, cm^−1^): 3392, 3062, 1841, 1762, 1705, 1494, 1479, 1454, 1397, 1359, 1339, 1299, 1220, 1200, 1158, 1101, 1066, 1036, 1026, 1012, 965.0, 956.6, 922.0, 889.1, 800.5, 772.6, 752.2, 719.4, 703.4, 669.1, 635.3, 622.9.

(p-Nitrophenyl)methyl 4-[3-(hydroxymethyl)-4-oxo-2-azetidinyl]-2-(imino)acetoacetate **9**.

To a solution of the acid chloride **8** (5.30 g, 10.4 mmol) in DCM (50 mL), at -78 °C under N_2_ was added dropwise to a solution of cold tetrabutylammonium borohydride (2.68 g, 10.4 mmol) in DCM (1 mL). The mixture was stirred at-78 °C for 3 min and then the reaction was quenched with TFA (1.60 mL, 4.82 mmol). The organic phase was washed with NH_4_Cl and brine, dried over Na_2_SO_4_, and concentrated to afford a viscous solid. The crude material was purified by flash chromatography (CH_3_OH: DCM, 0.5% to 20%) to give the titled compound **9** (2.09 g, 46 %). ^1^HNMR (400 MHz, CDCl_3_): δ 8.29 (d, 2H), 7.57 (d, 2H), 5.39 (s, 2H), 3.99 (d, 2H), 3.86 (dd, 1H), 3.60 (dd, 1H), 3.11 (m, 2H), 2.98 (m, 1H), 0.97 (s, 9H), 0.08 (d, 2H). ^13^C NMR (100 MHz, CDCl_3_): δ 191, 173, 172, 160, 148, 142, 129, 124, 65.6, 61.2, 60.9, 50.5, 48.8, 26.2, 18.4, 1.03, -5.29, -5.69. IR (KBr, cm^−1^): 3432, 2931, 2859, 2143, 1718, 1648, 1609, 1523, 1380, 1347, 1318, 1258.4, 1217.5, 1128, 996.1, 841.7, 807.9, 737.1, 681.1.

(p-Nitrophenyl)methyl 4-[3-(hydroxymethyl)-4-oxo-2-azetidinyl]-2-(imino)acetoacetate **10**

Alcohol **9** (14.9 g, 31.3 mmol) was dissolved in CH_3_CN (400 mL) and 15 mL of HF was added to the reaction mixture. Additional amount of HF (20 mL) was added to the reaction to complete. After the reaction completed, a finely ground powder of NaHCO_3_ was added to the mixture to adjust pH to 8. The solid was filtered and the filtrate was concentrated under reduced pressure to afford a white solid (11 g, 97%). ^1^H NMR (400 MHz, CDCl_3_): δ 8.22 (d, 2H), 7.55 (d, 2H), 6.90 (s, 1H), 5.25 (s, 2H), 4.10 (m, 3H), 3.06 (m, 2H), 2.90 (s,1H). ^13^C NMR (100 MHz, CDCl_3_): δ 190, 169, 161, 148, 142, 129, 124, 124, 65.6, 59.0, 58.7, 47.5, 44.9. IR (KBr, cm^−1^): 3362, 2947, 2143, 1720, 1648, 1608, 1522, 1386, 1347, 1296, 1216, 1129, 1015, 914.5, 853.3, 805.4, 740.1. HRMS (ESI-TOF): *m/z* calcd for C_15_H_14_N_4_O_7_ 385.0755, found 385.0747 [M+Na]^+^.

p-Nitrophenylmethyl 6-(hydroxymethyl)-3,7-dioxoazabicyclo[3.2.0]heptane-2-carboxylate **11**

Compound **10** (2.70 g, 7.42 mmol) was dissolved in EtOAc (45 mL). A catalytic amount of Rh_2_(OAc)_4_ (15 mg) was added to the reaction mixture. The reaction was refluxed to 50 °C for 1 h. The reaction was completed when bubbling subsided. The solvent was removed under reduced pressure to dryness. The entire compound was utilized directly for the next step. The yield was (2.40 g, 98%). ^1^H NMR (400 MHz, CDCl_3_): δ 8.27(d, 2H), 7.55 (d, 2H), 5.32 (dd, 2H), 4.81 (s, 1H), 4.10 (m, 2H), 3.46 (t, 1H), 2.98 (dd, 1H), 2.58 (dd, 1H). IR (KBr, cm^−1^): 3435.2, 2963, 1751, 1607, 1521, 1411, 1349, 1261, 1096, 799.4, 737.6, 661.0.

p-Nitrophenyl-3-{5-(dimethylamino)carbonyl-1-[(p-nitrophenyl)methoxycarbonyl]-3-pyrrolidinylthio}-6-(hydroxymethyl)-7-oxoazabicyclo[3.2.0]hept-2-ene-2-carboxylate **13**

Compound **11** (2.58 g, 7.72 mmol) was dissolved in CH_3_CN and cooled to -40 °C. Diphenyl phosphoryl chloride (1.59 mL, 7.72 mmol) was added first and then DIPEA (1.34 mL, 0.738 mmol) was added slowly to the reaction and stirred for 45 min. The reaction was monitored by ^1^H NMR. Upon completion of the reaction, thiol **12** (2.73 g, 7.72 mmol) and additional amounts of DIPEA (1.34 mL, 7.72 mmol) were added to the reaction and stirred for 1.5 h. Upon completion of the second half of the reaction, the solvent was removed and EtOAc was added to the residue and washed with NaHCO_3_, NH_4_Cl, dried over Na_2_SO_4_, and the solvent was removed under reduced pressure. The crude material was purified using column chromatography (DCM, MeOH as a gradient eluent from 0-10% MeOH). The yield was (400 mg, 15%). ^1^H NMR (400 MHz, CDCl_3_): δ 8.21 (dd, 2H), 7.51 (d, 2H), 7.31 (d, 2H), 7.22 (d, 2H), 5.30 (d, 2H), 5.24 (d, 2H), 4.70 (t, 1H), 4.04 (t, 1H), 3.36 (t,3H), 3.08 (d, 6H), 2.97 (t, 2H), 2.18 (t, 1H), 1.85 (t,2H). ^13^C NMR (100 MHz, CDCl_3_): δ 176, 171, 170, 161, 160, 154, 153, 147, 146, 144,143,142,128,127,124,123,76.7, 65.7,65.1, 61.9, 56.1,55.9, 53.8, 53.4, 53.1, 53.0, 41.4,40.8, 40.6, 36.9, 36.7, 36.0, 35.9, 21.76, 0.85. IR (KBr, cm^−1^): 3436, 3113, 3079, 2945, 2877, 1775, 1705, 1651, 1606, 1520, 1429, 1404, 1345, 1284, 1209, 1174, 1135, 1049, 1013, 857.0, 804.7, 766.7, 736.6. HRMS (ESI-TOF): *m/z* calcd for C_30_H_31_N_5_O_11_S 692.1633, found 692.1618 [M+Na]^+^.

3-{5-(Dimethylamino)carbonyl-1-[(p-nitrophenyl)methoxycarbonyl]-3-pyrrolidinylthio}-6-(hydroxymethyl)-7-oxoazabicyclo[3.2.0]hept-2-ene-2-carboxylic acid **MA-1-206**

Compound **13** (400 mg, 1.13 mmol) was added to a 50/50 mixture of EtOAc and pH = 6 buffer solution of sodium phosphate (pH was adjusted to 6 by using sodium hydroxide). 100 mg of 10% Pd/C was added to the mixture. The mixture was shaken on a Parr hydrogenator at 60 psi for 90 min. The slurry solution was then filtered through celite to remove the catalyst. The aqueous layer was separated and washed with mixture of ethyl acetate and ether, then concentrated to 10 mL and purified through an MCI gel CHP-20P, column eluting with (0-20%) ethanol/HPLC H_2_O. The yield was (8 mg, 2%). ^1^HNMR (400 MHz, CDCl_3_): δ 4.56 (t, 1H), 4.21 (t, 1H), 3.96 (m, 3H), 3.55 (m, 3H), 3.32 (m, 3H), 3.17 (s, 3H), 2.96 (s, 3H), 1.86 (d, 1H). HRMS (ESI-TOF): *m/z* calcd for C_15_H_21_N_3_O_5_S 356.1275, found 356.2720 [M+H]^+^.
