# Supplementary material for: C6 Hydroxymethyl-Substituted Carbapenem MA-1-206 Inhibits the Major Acinetobacter baumannii Carbapenemase OXA-23 by Impeding Deacylation
Source: mBio. 2022 Apr 14;13(3):e00367-22. doi: 10.1128/mbio.00367-22 (PMC9239083; doi:10.1128/mbio.00367-22)
Supplement: TABLE S1 [file mbio.00367-22-s0007.docx]

**Table S1. Time points used for the acylation of OXA-23 by MA-1-206**

| Time | Resolution (Å) | MA-1-206 occupancies | | MA-1-206 | Lys82^CO2^ | |
| --- | --- | --- | --- | --- | --- | --- |
|  |  | All atom average | O62-hydroxyl*^a^* | RSCC*^b^* | occupancy*^c^* | RSCC |
| 30 sec | 2.65 | 0.78 | 0.30 | 0.83 | 0.80 | 0.93 |
| 1 min | 2.45 | 0.82 | 0.53 | 0.83 | 0.86 | 0.94 |
| 2 min | 2.60 | 0.81 | 0.47 | 0.82 | 1.0 | 0.94 |
| 3 min | 2.40 | 0.88 | 0.88 | 0.87 | 0.96 | 0.94 |
| 5 min | 2.35 | 0.93 | 1.0 | 0.88 | 1.0 | 0.94 |
| 10 min | 2.35 | 0.97 | 0.97 | 0.91 | 1.0 | 0.98 |
| 25 min | 2.35 | 0.91 | 0.97 | 0.86 | 1.0 | 0.95 |

*^a^* Calculated with the occupancy for all MA-1-206 atoms, except C61 and O62, set at 1.0.

*^b^* Real-Space Correlation Coefficient calculated for the whole ligand.

*^c^* Calculated with all atoms of the lysine set to 1.0 except for the CX, OQ1, and OQ2 atoms of the carboxylate.
