# Supplementary material for: C6 Hydroxymethyl-Substituted Carbapenem MA-1-206 Inhibits the Major Acinetobacter baumannii Carbapenemase OXA-23 by Impeding Deacylation
Source: mBio. 2022 Apr 14;13(3):e00367-22. doi: 10.1128/mbio.00367-22 (PMC9239083; doi:10.1128/mbio.00367-22)
Supplement: TABLE S2 [file mbio.00367-22-s0008.docx]

**Table S2. OXA-23 MA-1-206 soak data collection statistics** *^a^*

|  | 30 s | 1 min | 2 min | 3 min | 5 min | 10 min | 25 min |
| --- | --- | --- | --- | --- | --- | --- | --- |
| Number of images | 600 | 600 | 600 | 600 | 1350 | 1125 | 1000 |
| Resolution range (Å) | 38.8-2.65  (2.78-2.65) | 38.9-2.45  (2.55-2.45) | 39.0-2.60  (2.72-2.60) | 39.0-2.40  (2.49-2.40) | 39.1-2.35  (2.43-2.35) | 39.2-2.35  (2.43-2.35) | 39.0-2.30  (2.38-2.30) |
| Reflections - obs. - unique | 160246  18257 (2378) | 201607  22984 (2545) | 170293  19299 (2278) | 217869  24682 (2532) | 520105  26213 (2525) | 439189  26505 (2463) | 411993  27628 (2636) |
| *R*_meas_ *^b^* | 9.2 (124.6) | 7.4 (127.0) | 11.0 (112.3) | 6.4 (95.5) | 12.8 (159.4) | 11.2 (159.3) | 7.6 (167.2) |
| *R*_pim_ *^b^* | 3.1 (41.6) | 2.5 (43.6) | 3.5 (36.2) | 2.1 (31.8) | 2.9 (37.6) | 2.8 (40.7) | 2.0 (42.8) |
| *I* / σ*I* | 13.4 (1.7) | 15.2 (1.6) | 9.5 (1.4) | 17.4 (2.0) | 13.0 (1.8) | 14.0 (1.9) | 18.3 (1.8) |
| Completeness (%) | 99.8 (99.1) | 100 (99.9) | 99.6 (98.9) | 99.9 (99.8) | 99.9 (99.5) | 99.5 (96.2) | 99.9 (99.1) |
| CC½ *^c^* | 0.999 (0.832) | 0.999 (0.801) | 0.998 (0.831) | 0.999 (0.893) | 0.997 (0.879) | 0.993 (0.868) | 0.999 (0.910) |
| Multiplicity | 8.8 (8.9) | 8.8 (8.3) | 8.8 (9.1) | 8.8 (8.8) | 19.8 (17.4) | 16.6 (14.8) | 14.9 (15.2) |
| Wilson B (Å^2^) | 83.7 | 75.1 | 86.7 | 69.7 | 64.1 | 69.2 | 64.0 |

*^a^* Numbers in parentheses refer to the highest resolution shell.

*^b^* R_meas_ is the redundancy-independent merging R factor. R_pim_ is the precision-indicating merging R factor (M. Weiss, *J. Appl. Crystallogr.* **34**, 130-135, 2001, https://doi.org/10.1107/S0021889800018227).

*^c^* Correlation between intensities from random half-sets of data (P.A. Karplus, K. Diederichs, *Science* **336**, 1030-1033, 2012, https://www.science.org/doi/10.1126/science.1218231).
