# Supplementary material for: C6 Hydroxymethyl-Substituted Carbapenem MA-1-206 Inhibits the Major Acinetobacter baumannii Carbapenemase OXA-23 by Impeding Deacylation
Source: mBio. 2022 Apr 14;13(3):e00367-22. doi: 10.1128/mbio.00367-22 (PMC9239083; doi:10.1128/mbio.00367-22)
Supplement: TABLE S3 [file mbio.00367-22-s0009.docx]

**Table S3. OXA-23 MA-1-206 soak refinement statistics**

|  | 30 s | 1 min | 2 min | 3 min | 5 min | 10 min | 25 min |
| --- | --- | --- | --- | --- | --- | --- | --- |
| Resolution (Å) | 38.8 – 2.65 | 38.9 – 2.45 | 39.0 – 2.55 | 39.0 – 2.40 | 38.3 – 2.35 | 38.7 – 2.35 | 39.0 – 2.30 |
| Reflections used – work/free | 18205/889 | 22946/1135 | 20377/1026 | 24647/1226 | 26167/1303 | 26478/1323 | 27559/1360 |
| *R*_work_ / *R*_free_ *^a^* | 21.77/25.32 | 23.89/26.67 | 24.14/26.52 | 22.81/26.00 | 24.17/26.83 | 22.41/24.80 | 24.29/27.28 |
| Number of atoms - protein  - MA-1-206  - water | 1918  24  22 | 1917  24  9 | 1918  24  - | 1918  24  17 | 1905  24  - | 1917  24  38 | 1911  24  12 |
| *B*-factors (Å^2^) - protein  - MA-1-206  - water | 88.2  91.6  75.5 | 90.4  96.8  78.9 | 89.2  92.2  - | 78.1  77.9  71.0 | 77.6  74.8  - | 74.0  77.7  65.0 | 82.6  78.9  64.4 |
| *rms* deviations - bonds (A)  - angles (°) | 0.008  1.13 | 0.008  1.16 | 0.009  1.17 | 0.008  1.11 | 0.008  1.14 | 0.009  1.13 | 0.009  1.08 |
| Ramachandran plot *^b^*  - residues in favored regions (%)  - number of outliers | 95.3  1 | 96.6  0 | 94.4  1 | 96.3  1 | 96.6  1 | 96.6  0 | 95.7  0 |

*^a^* R_free_ was calculated using a test set comprising 5% of the data.

*^b^* Calculated with the program MOLPROBITY (V.B. Chen *et al.*, *Acta Crystallogr.* **D66**, 12-12, 2010, https://doi.org/10.1107/S0907444909042073).
