# Supplementary material for: C6 Hydroxymethyl-Substituted Carbapenem MA-1-206 Inhibits the Major Acinetobacter baumannii Carbapenemase OXA-23 by Impeding Deacylation
Source: mBio. 2022 Apr 14;13(3):e00367-22. doi: 10.1128/mbio.00367-22 (PMC9239083; doi:10.1128/mbio.00367-22)
Supplement: TABLE S4 [file mbio.00367-22-s0010.docx]

**Table S4. OXA-23 imipenem soak data collection and refinement statistics *^a^***

|  | Imipenem-OXA-23 |
| --- | --- |
| *Data collection* |  |
| Resolution (Å) | 39.2-2.5 |
| Reflections: - observed  - unique | 370598  21845 (2411) |
| *R*_meas_ *^b^* | 8.3 (123.1) |
| *R*_pim_ *^b^* | 2.1 (29.4) |
| *I* / σ*I* | 20.3 (2.7) |
| Completeness (%) | 100 (100) |
| CC½ *^c^* | 0.999 (0.915) |
| Average multiplicity | 17.0 (17.4) |
| Wilson B (Å^2^) | 76.5 |
| *Refinement* |  |
| PDB Code | 7T7G |
| Resolution (Å) | 39.2 – 2.50 |
| Reflections used, work / free | 21832 / 1063 |
| *R*_work_ / *R*_free_ *^d^* | 25.68 / 29.15 |
| Number of atoms - protein  - imipenem  - water | 1911  20  13 |
| *B*-factors (Å^2^) - protein  - imipenem  - water | 83.2  84.2  60.4 |
| *rms* deviations - bonds (A)  - angles (°) | 0.008  1.09 |
| Ramachandran plot *^e^*  - residues in favored regions (%)  - number of outliers | 94.9  2 |

*^a^* Numbers in parentheses refer to the highest resolution shell (2.60 - 2.50 Å).

*^b^* R_meas_ is the redundancy-independent merging R factor. R_pim_ is the precision-indicating merging R factor (M. Weiss, *J. Appl. Crystallogr.* **34**, 130-135, 2001, https://doi.org/10.1107/S0021889800018227).

*^c^* Correlation between intensities from random half-sets of data (P.A. Karplus, K. Diederichs, *Science* **336**, 1030-1033, 2012, https://www.science.org/doi/10.1126/science.1218231).

*^d^* R_free_ was calculated using a test set comprising 5% of the data.

*^e^* Calculated with the program MOLPROBITY (V.B. Chen *et al.*, *Acta Crystallogr.* **D66**, 12-12, 2010, https://doi.org/10.1107/S0907444909042073).
